# A refined proposal for the origin of dogs: the case study of Gnirshöhle, a Magdalenian cave site

**DOI:** 10.1038/s41598-021-83719-7

**Published:** 2021-03-04

**Authors:** Chris Baumann, Saskia Pfrengle, Susanne C. Münzel, Martyna Molak, Tatiana R. Feuerborn, Abagail Breidenstein, Ella Reiter, Gerd Albrecht, Claus-Joachim Kind, Christian Verjux, Charlotte Leduc, Nicholas J. Conard, Dorothée G. Drucker, Liane Giemsch, Olaf Thalmann, Hervé Bocherens, Verena J. Schuenemann

**Affiliations:** 1 Biogeology, Department of Geosciences, University of Tübingen, Hölderlinstraße 12, 72074 Tübingen, Germany; 2 Institute for Archaeological Sciences, University of Tübingen, Rümelinstraße 23, 72070 Tübingen, Germany; 3 Institute of Evolutionary Medicine, University of Zurich, Winterthurerstrasse 190, 8057 Zurich, Switzerland; 4 Centre of New Technologies, University of Warsaw, S. Banacha 2c, 02-097 Warsaw, Poland; 5 Section for Evolutionary Genomics, GLOBE Institute, Øster Farimagsgade 5, Bygning 7, 1353 København K, Denmark; 6 Department of Archaeology, Markgräflerland-Museum Society, Wilhelmstraße 7, 79379 Müllheim, Germany; 7 State Office for Cultural Heritage Baden-Württemberg, Berliner Str. 12, 73728 Esslingen, Germany; 8 Service Régional de l’Archéologie (UMR 7041 ArScAn-Équipe Ethnologie Préhistorique), DRAC Centre, Val de Loire, 6 Rue de la Manufacture, 45000 Orléans, France; 9 INRAP, 12 Rue de Méric, 57000 Metz, France; 10 UMR8215-Trajectoires, CNRS, 21 Allée de l’Université, 92023 Nanterre Cedex, France; 11 Department for Early Prehistory and Quaternary Ecology, University of Tübingen, Burgsteige 11, 72070 Tübingen, Germany; 12 Senckenberg Centre for Human Evolution and Paleoenvironment, Schloss Hohentübingen, University of Tübingen, 72070 Tübingen, Germany; 13 Senckenberg Centre for Human Evolution and Palaeoenvironment, University of Tübingen, Sigwartstraße 10, 72076 Tübingen, Germany; 14 Archäologisches Museum Frankfurt, Karmelitergasse 1, 60311 Frankfurt am Main, Germany; 15 Department of Pediatric Gastroenterology and Metabolic Diseases, Poznan University of Medical Sciences, Szpitalna 27/33, 60-572 Poznan, Poland; 16 Senckenberg Centre for Human Evolution and Palaeoenvironment, University of Tübingen, Rümelinstraße 23, 72070 Tübingen, Germany

**Keywords:** Ecology, Evolution, Genetics, Ecology

## Abstract

Dogs are known to be the oldest animals domesticated by humans. Although many studies have examined wolf domestication, the geographic and temporal origin of this process is still being debated. To address this issue, our study sheds new light on the early stages of wolf domestication during the Magdalenian period (16–14 ka cal BP) in the Hegau Jura region (Southwestern Germany and Switzerland). By combining morphology, genetics, and isotopes, our multidisciplinary approach helps to evaluate alternate processes driving the early phases of domestication. The isotope analysis uncovered a restricted, low *δ*^15^N protein diet for all analyzed Gnirshöhle specimens, while morphological examinations and phylogenetic relationships did not unequivocally assign them to one or the other canid lineage. Intriguingly, the newly generated mitochondrial canid genomes span the entire genetic diversity of modern dogs and wolves. Such high mitochondrial diversity could imply that Magdalenian people tamed and reared animals originating from different wolf lineages. We discuss our results in light of three ecological hypotheses and conclude that both domestication and the existence of a specialized wolf ecomorph are highly probable. However, due to their proximity to humans and a restricted diet, we propose domestication as the most likely scenario explaining the patterns observed herein.

## Introduction

In line with several theories detailing biological diversity and evolution, the survival of species is often predicated on their ability to adapt and thrive within a changing environment. To do so, plant and animal species have developed several strategies, including the adaptation to existing, or the development of new ecological niches^1^. This is exemplified by various studies that have investigated faunal adaptation to the environment before and after the Last Glacial Maximum (LGM), a period of climatic fluctuation and dramatic landscape changes^2^. One possibility to survive unfavorable conditions is to retreat into *refugia,* i.e. restricted and often isolated areas still harboring ecological and environmental features beneficial for the species in question^3,4^. Specifically, during the LGM, various species retreated to warmer areas such as the Iberian Peninsula^5^, subsequently preserving and shaping patterns of genetic variation prevalent during this epoch^6^.

An alternative pathway for survival is to form so-called ecomorphs and thus adapt to environmental conditions and habitats^7^. Ecomorphs are characterized by a genetic variety within a species or a variety of several species exposing the same phenotypic features, due to the adaptation to a local ecology. One prominent example of a species represented by many ecomorphs since prehistoric periods is the grey wolf (*Canis lupus*). The wolf is a mobile carnivore with an opportunistic diet^8^, and can occupy different trophic niches, best exemplified by Late Pleistocene wolves, which were characterized by two different dietary habits^9,10^.

During the Late Pleistocene, as humans became more numerous and intrusive^11–14^, they started to re-shape their local environment and thereby became a driving factor within the landscape providing artificial ecological and dietary (= trophic) niches^15–17^. The concept of humans as niche constructors is vividly debated and recently contributed to our understanding of domestication^18,19^. This debate includes the origin of modern dogs, which may have occurred intentionally by pet-keeping, a side-effect of goal-orientated domestication, or unintentionally (self-domestication), when humans created a niche for commensal scavengers with their food waste. The uniting element of these theories is the subsequent selection for tameness and reduced fear, resulting in decreased wariness and aggression, high tolerance of penning, and sexual precocity^20,21^.

Although the when and where of wolf domestication are still disputed, it is now accepted that dogs are indeed the oldest domesticates^10,21–24^. Once the process of domestication began, humans quickly gained control over the diet, reproduction, and health of their new companions and thus set the stage for a lasting human–dog bond^21,24,25^. For example, stable isotope analyses of canids found in Předmostí, a Gravettian site in the Czech Republic dated to 31,500 years ago, showed evidence of adaptation to two different dietary niches^21,26,27^; however, it remains unclear whether or not one of these two different canid groups are indeed early domesticated wolves^24,27,28^. While such older dates for potential onsets of wolf domestication have been challenged, it is widely accepted that 16,000 years ago is the approximate time for the first emergence of dogs^10^. From this time onwards, dog remains have been discovered at several archaeological sites throughout Europe, such as the famous dog from Bonn-Oberkassel excavated alongside two human burials^25,29,30^. Another important site with possible early dog remains is Kesslerloch^31,32^ (Canton Schaffhausen) located in the Hegau Jura and dating to the late Magdalenian period (17.5–14.3 ka cal BP^33^). Morphological examinations of these remains revealed a distinction between wolves and dogs^32,33^ and dietary reconstruction analyses^16^ demonstrated parallel occurrences of two distinct canid trophic niches at this site. However, it remains unclear whether Kesslerloch is a unique case or if wolf domestication was practiced in the entire region over a longer period of time. The Hegau Jura, including the Kesslerloch site and two other cave sites, Petersfels and Drexlerloch, represents a Magdalenian hotspot for human activity in the pre-Alpine region. In the heart of this setting lies the cave site Gnirshöhle, which also shows evidence of human occupation, with butchered animal bones, worked antlers, and bone needles^34^ (Supplementary Note 1), as well as provides a cohort of large canid remains, making this cave particularly valuable for investigating pre-Alpine canids.

**Figure 1:**
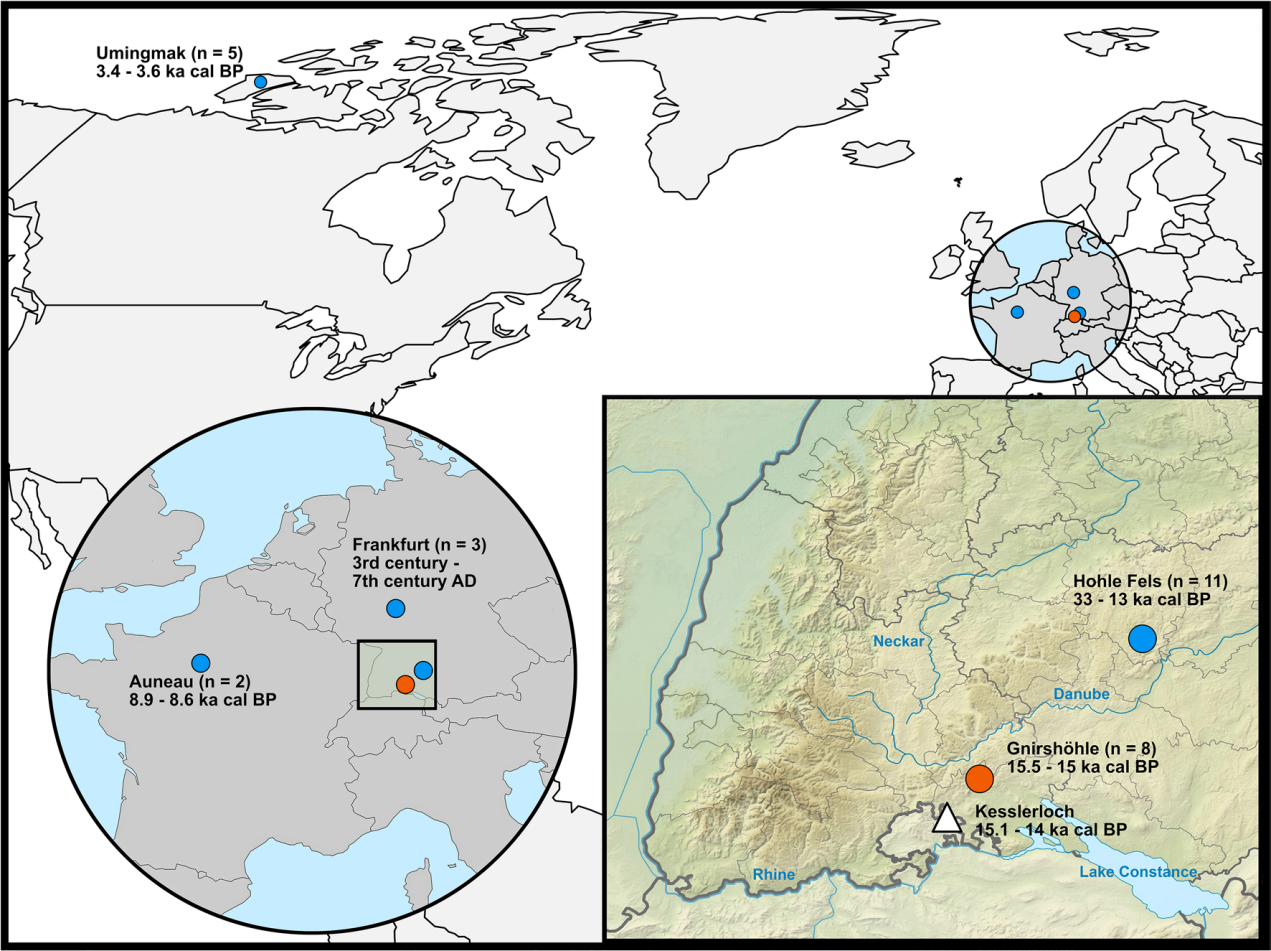
Map of the archaeological sites from which the canid remains were retrieved and investigated. Dots represent the sites from which new data are included in this study, the triangle marks a site with important comparative data. Additional information about sample sizes and the archaeological dates are provided in Table S1. Dates are given in ka (kilo annos) cal BP, for samples younger than 2000 years dates are given in century AD.

In the present study, we applied a multidisciplinary approach to study canid remains from the Gnirshöhle and assessed canid population dynamics potentially shaped by environmental conditions and anthropogenic pressure during the Magdalenian in the Hegau Jura (Fig. 1). By combining isotopic dietary reconstruction, metric assessment, and paleogenetics, we were able to test various hypotheses to better elucidate the onset of wolf to dog transition in the Hegau Jura region and derived a refined proposal of wolf domestication.

## Results

### Archaeozoology: morphological and metric results

All samples except one (GN-999) were assigned to be large canid specimens (*Canis* sp.) by archaeozoological classification. The mandible GN-999 (Figure S2) was described earlier as a wolf-like specimen with morphological traits of domestication^34^. The mandible is relatively short and exhibits tooth crowding between P_4_ and M_1_. GN-999 contains a tooth row with P_2_, P_3_, P_4_, M_1_, and M_2_, while P_1_ is missing and M_3_ was lost premortem; the alveolus of these two missing teeth is still visible.

**Figure 2:**
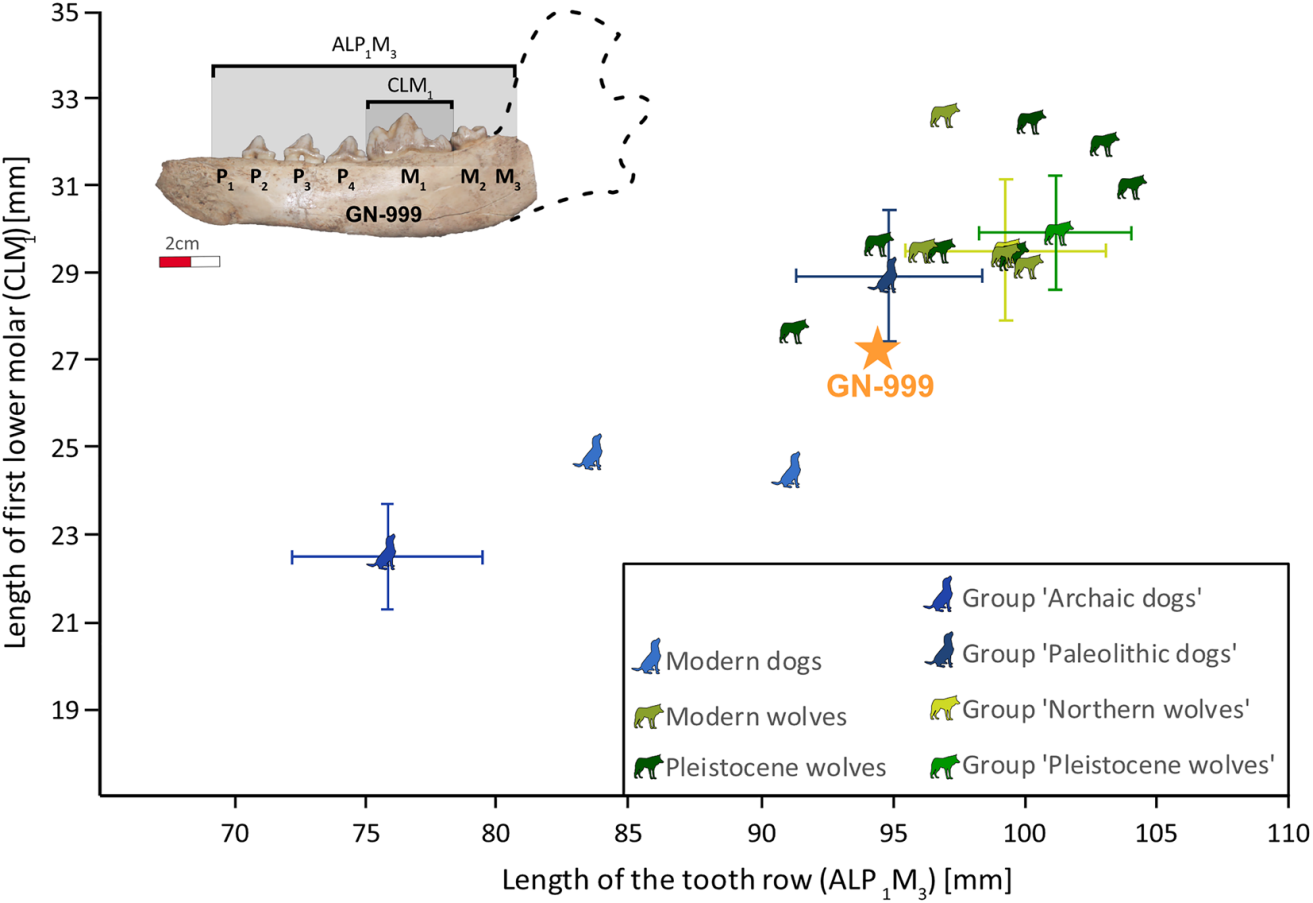
Metric data of GN-999 (black star) in comparison to metric data of seven canid groups (see Table S2): Modern dogs (n = 2, reference collection Uni Tübingen); Modern wolves (n = 4, reference collection Uni Tübingen and Bonn); Pleistocene wolves (n = 7, Brillenhöhle, Kesslerloch, Geißenklösterle. The four groups ‘Archaic dogs’ (n = 27), ‘Palaeolithic dogs’ (n = 31), ‘Northern wolves’ (n = 35) and ‘Pleistocene wolves’ (n = 36) are defined by Germonpré and colleagues^35^ and defined by mean value and standard deviation.

The length of the tooth row of the mandible GN-999 (ALP_1_M_3_ = 94.4 mm) plotted against the length of the first molar (CLM_1_ = 27.3 mm) falls into the observed absolute ranges of recent Northern wolves and ‘Palaeolithic dog’ group^35^. Although, GN-999 is considerably shorter by one standard deviation than the lower bound of the range for modern and Pleistocene wolves, as well as the ‘Paleolithic dog’ group^35^. This specimen is also morphologically similar to a small wolf from Kesslerloch^32^ (Fig. 2, Table S4). Therefore, we cannot exclude GN-999 assigned as a wolf using metric data (other standardized measurements after von den Driesch^36^ concerning GN-999 and the maxilla GN-192 are given in Supplementary Note 2 and Tables S2 and S3). Tooth crowding of the premolars is a typical trait for dogs and is related to shortening of the snout during the process of domestication^37^. However, crowding between P_4_ and M_1_ is also observed in wolves^38^. Additionally, a diastema is present between the P_2_ and the P_3_, which is rather typical for recent and Pleistocene wolves^35^. Thus, the morphological and metric assignments for GN-999 remain unresolved.

### Stable isotope analysis: niche modeling and dietary reconstruction

**Figure 3:**
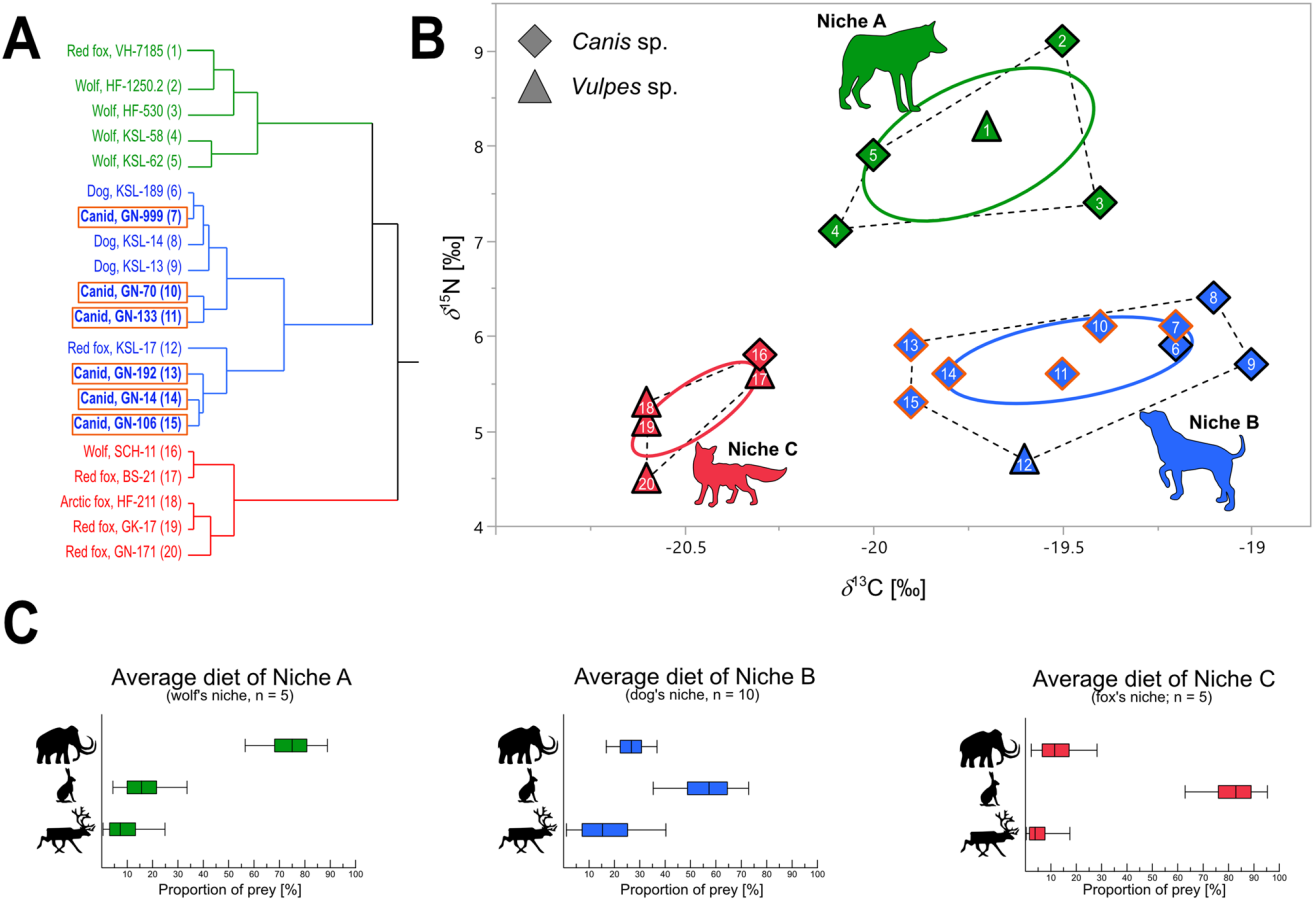
**(A)** Cluster analysis of stable isotopic values of the canids. **(B)** Reconstructed trophic niches and **(C)** dietary reconstruction of the ancient Magdalenian canids based on stable isotope data of carbon and nitrogen preserved in bone collagen. Labels of the new samples from this study are in bold and marked with an orange rectangular frame. Numbers in **(B)** correspond to these in **(A)**.

The percentage of nitrogen in bone was measured for a total of ten canids (Table S1 and Supplementary Note 4); only six of the Gnirshöhle canids, the red fox from Bockstein, and the pre-LGM wolf from Hohle Fels had sufficient collagen content for analysis (1.1–3.1% N_bone_)^39^. Specimen HF-912 was removed from the isospace published by Baumann and colleagues^16^, as it was dated to the pre-LGM (Supplementary Note 3). The newly analyzed HF-1712 sample, contextually dated to pre-LGM, was also excluded. The slightly younger specimen, HF-1250.2 (Supplementary Note 3), was dated to 11,400 ± 30 BP (13,308–13,150 cal BP), and therefore belonged to the Late Palaeolithic period. However, since both archaeological periods are temporarily close and the environmental conditions did not change dramatically, we decided to keep the isotopic values of this specimen in the reconstructed Magdalenian isospace. We calculated three canid niches: niche A, niche B, and niche C (Fig. 3A,B), in accordance to the *δ*^13^C and *δ*^15^N values of each specimen. For niche A and C, we observed no changes compared to the previously published isospace^16^. Niche B, however, is further extended with respect to the *δ*^13^C values. Briefly, five wolves^16^ from Kesslerloch and Hohle Fels, as well as one red fox^16^ from Vogelherd fell into niche A (Fig. 3A), with *δ*^13^C values ranging from − 20.1 to − 19.4‰ and *δ*^15^N values ranging from + 7.1 to + 9.1‰. Niche B included three dogs^16,40^, one red fox^40^ from Kesslerloch, and all newly analyzed canids from Gnirshöhle (Fig. 3A). The isotopic values of this niche ranged from − 20.0 to − 19.0‰ and from + 4.7 to + 6.4‰ for *δ*^13^C and *δ*^15^N, respectively. Finally, niche C included three red foxes^16^ from Geißenklösterle, Gnirshöhle, and Bockstein, one arctic fox^16^ from Hohle Fels, and one wolf^40^ from Schussenquelle (Fig. 3A). This niche is characterized by *δ*^13^C values ranging from − 20.6 to − 20.3‰ and *δ*^15^N values ranging from + 4.5 to + 5.8‰.

We reconstructed the percentages of three different dietary sources for each niche (i.e., megafauna, small game, ungulates; see methods section, and Table S5), using the Bayesian statistic model MixSIAR. The dietary preferences strongly varied among the niches (Fig. 3C, Table 1). Specimens of niche A had a preference for megaherbivores, such as mammoths. Members of niche B fed on small mammals, such as hares, and in addition, on ungulates, such as reindeer and horse, and megaherbivores. Lastly, individuals in niche C had a high preference for small mammals.

**Table 1:**
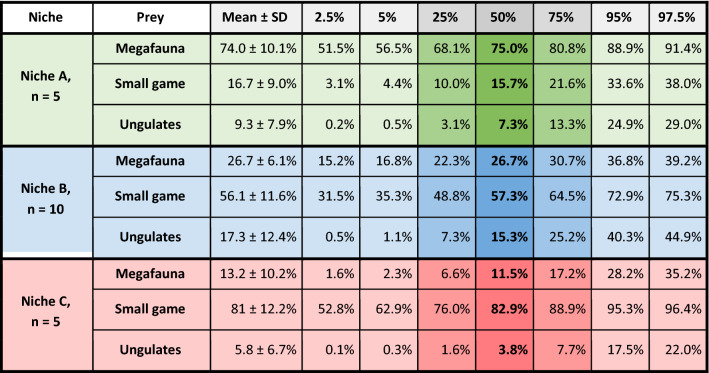
Contribution of prey types to the three reconstructed trophic niches.

### Palaeogenetics: phylogeny, genetic diversity, and evolutionary trajectory

Although our genetic investigations were focused on analyzing the mitochondrial DNA sequences of the canid samples from Gnirshöhle (n = 8), additional mitochondrial genomes from canid remains of diverse temporal and geographic origin (Fig. 1, Supplementary Note 1, Table S1) were also generated and included to increase the genetic diversity. Out of 28 extracted samples, we were able to reconstruct a total of 23 (77%) complete or nearly complete mitochondrial genomes, including five Magdalenian mitochondrial genomes from Gnirshöhle, and one from Hohle Fels (Table S1). The mean coverage ranged from 7.0 to 341.2 (Table 2), and all reconstructed mitochondrial genomes had a threefold coverage between 93.5 and 100% of the ~ 16,000 mitochondrial nucleotides (Table 2).

**Table 2:** Results of the genetic analysis.

Short ID	Site	Period	Genetic ID	Mapped reads after RMDup	Endogenous DNA (%)	Mean coverage	Coverage ≥ 3× in %	DMG 1st base 3'	DMG 1st base 5'
GN-999	Gnirshöhle	Magdalenian	TU859	5055	0.40	16.60	98.60	0.29	0.31
GN-14	Gnirshöhle	Magdalenian	TU1077	1647	0.29	7.28	94.83	0.62	0.62
GN-106	Gnirshöhle	Magdalenian	TU1078	1748	0.28	7.00	93.50	0.66	0.62
GN-133	Gnirshöhle	Magdalenian	TU1072	4150	0.84	17.83	97.92	0.33	0.36
GN-192	Gnirshöhle	Magdalenian	TU1073	8231	1.63	31.51	98.05	0.35	0.36
HF-1250.1	Hohle Fels	*Collapsed profile*	JK2176	23702	8.51	97.21	99.64	0.37	0.37
HF-1250.2	Hohle Fels	Late Palaeolithic	JK2179	28787	5.72	129.31	99.97	0.29	0.29
HF-912	Hohle Fels	Gravettian	JK2177	2789	0.28	7.77	92.49	0.35	0.35
HF-530	Hohle Fels	Magdalenian	JK2181	86893	12.53	341.28	99.99	0.38	0.39
HF-1965	Hohle Fels	Gravettian	JK2174	31838	6.88	183.47	99.95	0.22	0.23
HF-1390	Hohle Fels	Gravettian	JK2183	33157	12.93	230.55	100.00	0.21	0.21
HF-1174	Hohle Fels	Gravettian	JK2178	10320	1.29	35.73	98.87	0.38	0.37
HF-1712	Hohle Fels	Grav/Aurig	JK2182	16492	1.78	61.14	98.85	0.33	0.34
HF-1035	Hohle Fels	Aurignacian	JK2175	12568	0.64	49.29	98.93	0.28	0.27
HF-1553	Hohle Fels	Aurignacian	JK2180	3856	0.43	12.30	97.13	0.37	0.40
APC-19	Auneau	Mesolithic	TU839	24895	5.07	93.72	99.62	0.33	0.42
APC-20	Auneau	Mesolithic	TU840	25267	3.61	101.02	99.39	0.22	0.33
UA-205A	Umingmak	Palaeoeskimo	TU148	31662	12.29	170.10	99.83	0.09	0.10
UA-206	Umingmak	Palaeoeskimo	TU144	20637	15.11	89.23	99.84	0.06	0.07
UA-207	Umingmak	Palaeoeskimo	TU146	19170	6.68	86.30	99.91	0.04	0.03
UA-208	Umingmak	Palaeoeskimo	TU145	15976	3.82	62.17	99.70	0.06	0.07
F-1986.1	Frankfurt	Merovingian	TU387	23908	3.81	98.79	99.13	0.17	0.31
F-1986.2	Frankfurt	Merovingian	TU389	26400	4.72	108.05	99.56	0.20	0.30
F-α19496	Frankfurt	Roman	TU390	27380	4.68	110.47	98.79	0.21	0.32

Table represents the number of mapped reads against the reference genome (after duplicate reads removal), endogenous DNA content, the percentage of mitochondrial genome bases covered at least 3-fold and the fraction of 1st base damage at the 3' and 5' end of the mapped sequences reads. HF-1250.1 and HF-1250.2 were concluded as one individual according to genetic and archaeological analysis and were merged into one sample for downstream analyses.

**Figure 4:**
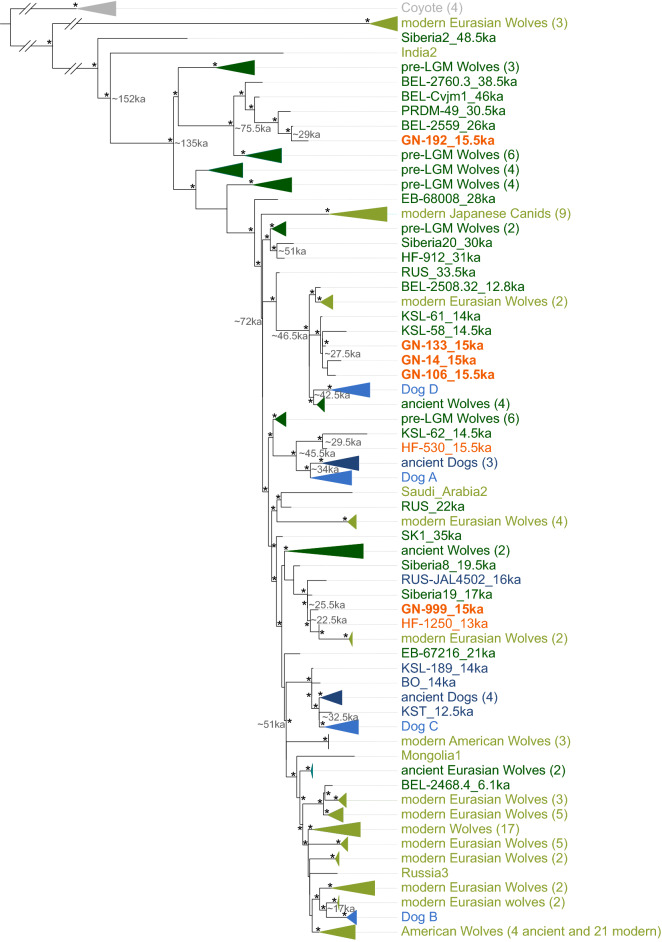
Maximum likelihood (ML) consensus tree of mitochondrial canid genomes. Newly generated samples are highlighted in orange; with the GN canids further bolded. Statistical support was assessed by generating 10,000 bootstrap replicates and nodes with bootstrap support higher than 95% are shown with an asterisk. Important nodes are labeled with respective node ages (given in ka cal BP) estimated using time-aware Bayesian phylogenetic inference (Figure S7). Ancient dogs are labeled in dark blue, modern dogs in light blue. Ancient wolves in dark green, modern wolves in light green. The ages of samples older 1,000 years are given in ka (cal BP).

The phylogenetic arrangement, estimated in a Maximum Likelihood framework (Fig. 4), did not reveal any clear chronological or spatial differentiation of our six Magdalenian samples compared to the assemblage of ancient and modern canids. Our novel Magdalenian mitochondrial genomes clustered with ancient mitochondrial genomes from Belgium, the Czech Republic, and Russia, ranging in age from the pre-LGM (ca. 50—28 ka cal BP) to the post-LGM (19.5—16 ka cal BP), in addition to modern canids of global origins (Fig. 4). Interestingly, one mitochondrial genome, from the Magdalenian specimen GN-192 (ca. 15.6—15.2 ka cal BP, Supplementary Note 3, Figure S1), fell within a very basal cluster that was previously assigned exclusively to Belgian pre-LGM canids. Taking the age of this specimen into account (Supplementary Note 3), this finding implies a genetic continuity of one maternal canid lineage from the pre-LGM to the Magdalenian. Furthermore, the specimens GN-14, GN-106, and GN-133 are placed closely to the two ancient wolves KSL-58^25^ and KSL-61^41^; additionally, the specimen HF-530 is placed with a third Swiss mitochondrial genome, KSL-62 (Fig. 4).

Altogether, these results demonstrate a close maternal relationship of temporally spaced specimens from the same region in southwestern Germany. Despite the close relationship of these specimens to others within the region, we also observed a close genetic affinity of temporally and geographically distantly spaced maternal haplotypes, e.g., GN-999 was placed close to mitochondrial DNA sequences of ancient and modern canids originating from northeastern Europe, Siberia, and eastern Beringia. In general, we observed that our reconstructed mitochondrial genomes for specimens older than the Mesolithic, an archaeological period of the early Holocene, are maternally more closely related to modern and ancient wolves than to modern and ancient dogs (Figure S5). Nevertheless, an assignment of the canids as dogs or wolves cannot be unambiguously performed based solely on the genetic affinity.

As evidenced by the broad distribution of our samples throughout the phylogeny, the Magdalenian canids from southwestern Germany harbor a vast amount of genetic diversity. To assess the genetic diversity of the Magdalenian Gnirshöhle canids, we compared the nucleotide diversity (π) of those specimens with the observed nucleotide diversity of the Kesslerloch canids, in addition to modern dogs and wolves sampled worldwide (Tables S7, S8, Figure S7). Nucleotide diversity is defined as the average number of differences per site between any two DNA sequences in the population^42^, and is less biased by sample size and potential sequencing errors than, for instance, haplotype diversity. The estimated nucleotide diversity of our five novel sequences from the Gnirshöhle canids (n = 5; π = 0.00306 ± 0.00106) was almost as high as that observed in a global assemblage of modern dogs (n = 79; π = 0.00339 ± 0.00021) and slightly lower than the diversity determined for the Kesslerloch canids (n = 5; π = 0.003379 ± 0.00098). These estimates of genetic diversity illustrate the high genetic variation in Magdalenian canids originated from caves located in the Hegau Jura region.

To evaluate the evolutionary timescale of the canid mitochondrial phylogeny, we performed BEAST analyses (Figure S6). The results produced a very similar topology compared to the Maximum Likelihood tree (Figure S5), except for minor differences whenever the statistical support was low, as for example the dogs within clade A, or amongst North American wolves. As part of the phylogenetic timescale estimation, the ages of the samples not directly dated by ^14^C analyses were estimated. While the median ages for most samples do not differ substantially from their respective archaeological age (Table S6), the age of the three canid samples from Frankfurt did; for F-1986.2 approximately 4 ka cal BP, for F-1986.1 and F-α19496 about 6 ka cal BP. However, the archaeological ages of these samples fall within the 95% credibility intervals of the age estimates and the departure of median estimated age is likely caused by the young age of the samples being close to the minimum age constraint (0 ya) of the wide priors (uniform 0–100 ka) for the age parameter. Interestingly, for HF-912, the estimated age (~ 42 ka BP) was older than expected considering the sample was excavated from a layer contextually dated to the Magdalenian. This finding agrees with the taphonomic observations concerning the bone color that suggested the sample may be older than the layer in which it was found and has been additionally confirmed by ^14^C dating (31.4 ka cal BP, Supplementary Note 3).

To further elucidate the dynamics of wolf domestication, we estimated the timescale of the inferred canid mitogenomic evolutionary tree. As outlined by Loog and colleagues^41^, the coyote outgroup and the modern wolf samples from the Himalayas were excluded from this analysis. The TMRCA of all dogs and wolves was estimated to approximately 152 ka cal BP (111–231 cal ka BP 95% HPD; Fig. 4). The TMRCA for clade A was estimated to around 34 ka cal BP, clade B to circa 6.5 ka cal BP, clade C to approximately 22.5 ka cal BP, and clade D to about 3.5 ka cal BP. Our six novel Magdalenian mitogenomes fall outside these clades, but four are genetically close to either clade A or D, showing a presence of close relatives to modern dogs in the Magdalenian Hegau Jura. On the other hand, the same small region and period also hosted very genetically distant canids that diverged with modern dog lineages as early as 135 ka cal BP.

## Discussion

Most studies focusing on ancient canids and the proposal of various scenarios for potential wolf domestication have previously used one line of evidence, such as morphological examinations, isotopic analyses, or genetic investigations^21,22,25–27,35,41^. Hence, studies analyzing the same archaeological material often lacked a consensus and often resulted in divergent outcomes^25,26,35,41^. Our multidisciplinary approach combined various archaeological disciplines to analyze pre-Alpine Magdalenian canids and revealed high mitochondrial variation prevalent in a geographically restricted area—the Gnirshöhle (Fig. 4). While Thalmann and colleagues^25^ had observed high variation among Pleistocene canids in general, our findings now highlight the Hegau Jura region, encompassing Magdalenian caves such as Gnirshöhle and Kesslerloch, as a hot-spot for genetic variation in Pleistocene canids. Interestingly, the variation within the Gnirshöhle mirrors the collective variation of almost all canids analyzed herein.

Utilizing one of the most comprehensive assemblages of canid mitochondrial genomes varying in time and space, we were able to associate dog and wolf haplotypes of modern and ancient origin and infer the time to their common ancestor. Intriguingly, the large data assemblage enabled us to genetically assign the Kesslerloch specimen KSL-189 as dog-like in contrast to the study of Loog and colleagues^41^, in which the canid was included in genetic examination of ancient wolves. Due to the close genetic relatedness to the Bonn-Oberkassel canid, a widely accepted early dog, combined with dietary analysis^16^ (Fig. 2A,B) and morphological study^32^, we revised the status of KSL-189 as dog-like.

In general, our TMRCA estimates are in line with previous findings placing a common ancestor of dogs and wolves in the late Pleistocene (oldest clade dog A—wolf: ~ 46 ka BP^24,25,43^). It should be emphasized that the TMRCA is not equivalent to a population split^44^, nor does it represent the actual onset of domestication; it can, however, be used to assess an upper time limit of such events. We attribute slight discrepancies between the previously published TRMCAs and our new estimates to varying sampling regimes and parameter settings used in the different studies^24,25,41,43^. The most recent common ancestor of all closely contemporaneous samples from Gnirshöhle dates back to 135 ka BP (including GN-192). This is an intriguing finding for several reasons. First, Magdalenian mitochondrial genomes reconstructed from specimens originating from a single cave in southwestern Germany captured almost the entire breadth of genetic diversity of all contemporary and ancient dogs and most wolves. Second, the newly generated mitochondrial genomes from the Gnirshöhle canids introduce a yet unrecognized, ancient mitochondrial canid lineage that had survived into the Magdalenian. Several authors have now proposed that mitochondrial dog lineages in prehistoric Europe were replaced by expanding lineages arriving from the East^24,45,46^. This fate was possibly shared by the Gnirshöhle canids as an example of diversity that was replaced by the incoming lineages represented by today's dog clade A^43^. The age of the Gnirshöhle canids exceeds those of the samples analyzed by Frantz and colleagues^24^, and implies that with more samples, we may still discover divergent lineages representative of early dogs^47^, a prospect that helps to finally derive a more nuanced picture of modern wolf’s domestication history (see also Bergström and colleagues^46^).

**Figure 5:**
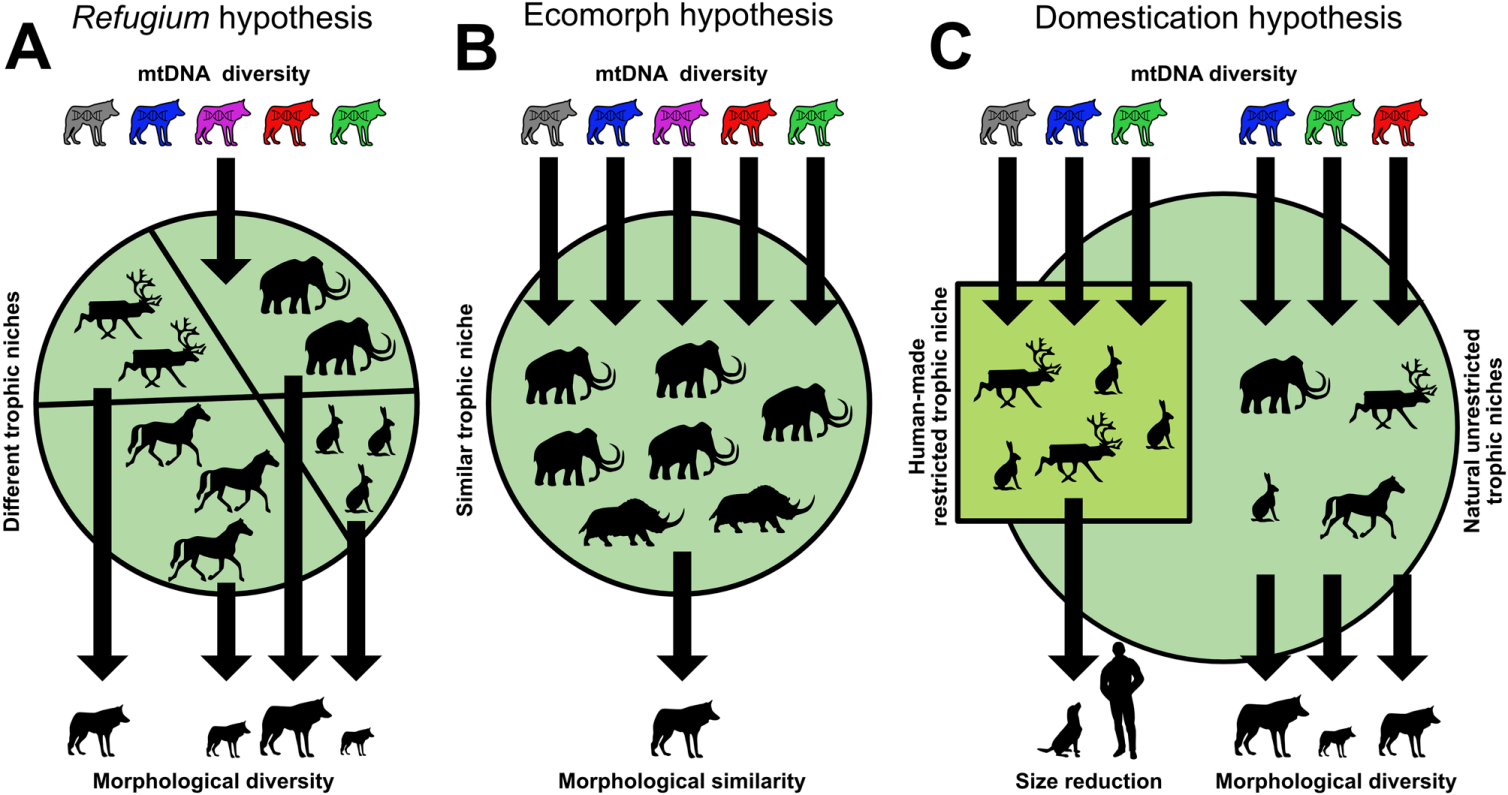
Graphic presentation of the three hypotheses of the adaptation to environmental changes; **(A)** *refugium* hypothesis, **(B)** ecomorph hypothesis, **(C)** domestication hypothesis.

To better explain the observed genetic diversity, we expanded our focus to include niche and dietary reconstructions to assess the trophic behavior, as well as a comparison with two other Magdalenian cave sites, Hohle Fels and Kesslerloch, located less than 100 km away. With respect to their isospace, all specimens from the Gnirshöhle were assigned to the trophic niche B that also includes the Kesslerloch dogs^16^, a finding that is in contrast to the detected closer genetic affinity of the Gnirshöhle canids to modern and ancient wolves (Fig. 4) and warrants further explanation. Carbon and nitrogen isotopes in bone collagen accumulate over several years before tissue turnover occurs^48–50^, therefore, tissue samples rather than indicating seasonal or single dietary events reflect a food resource that would have been regularly available over a long period of time. The newly generated data allowed us to test three hypotheses explaining the diversity patterns in Magdalenian canids from the Hegau Jura: (1) the *refugium* hypothesis, (2) the ecomorph hypothesis, and 3) the domestication hypothesis (Fig. 5).

Previous studies of genetic diversity in *refugia* have shown that a direct correlation exists between the size of the *refugium* and the observed diversity (e.g.,^51^). The larger an area, the more genetic variation could have accumulated consequently resulting in higher estimates of the genetic diversity of the species under consideration. If we accept the Hegau Jura region as a potential canid *refugium*, we should observe lower diversity in the mitochondrial genomes, as it is rather small compared to, for instance, the Iberian Peninsula *refugium*^5,51,52^. Furthermore, a retreat into glacial *refugia* would not necessarily require a shift in the trophic niche of the focal species, and hence under the *refugium* hypothesis, we would predict a diet composition similar to that of pre-LGM canids from southwestern Germany. Results of dietary reconstructions from the pre-LGM periods of the Swabian Jura^17^ have shown that all studied canids belonged exclusively to one group with high *δ*^15^N values that primarily fed on megafauna. While we observed the same pattern in the Magdalenian ‘wolf’ niche (niche A), canids from Gnirshöhle behaved differently, which led us to reject the *refugium* hypothesis.

With regard to the ecomorph and domestication hypotheses, we are not able to unequivocally differentiate between either solely based on our mitochondrial DNA data. Both hypotheses are equally supported by the high genetic diversity within the Magdalenian canids in addition to its dissociation from space and time. This demonstrates the limitation of genetic analysis of the maternally inherited mitochondrial genome to appropriately examine such complex hypotheses. From the archaeozoological perspective, neither the metrics nor tooth crowding^38,53^ was sufficient to differentiate whether the canids of Gnirshöhle were dogs or wolves.

Considering the trophic niche observed among the Gnirshöhle canids being defined as a Late Pleistocene ecomorph and bearing in mind the properties of a surviving wolf ecomorph from the pre-LGM, we predict a restriction to a similar diet, as was observed for an Eastern Beringia Pleistocene ecomorph^9^. All Gnirshöhle canids showed signals indicating they consumed a low *δ*^15^N protein diet (niche B), while those from the two nearby cave sites, Kesslerloch and Hohle Fels, showed a high *δ*^15^N protein dietary source like those consumed by other canids^16^. Thus it is unlikely that the Gnirshöhle canids represent a surviving wolf ecomorph with a similar high *δ*^15^N protein diet as the pre-LGM wolves^17^. However, it is possible that canids consumed reindeer and small game and may be defined by a new type of ecomorph, similar to the hypothesis proposed by Perri^10^ concerning canids from the Gravettian open-air site Předmostí (Czech Republic). Members of the Magdalenian niches A and B show similar reconstructed diets compared to those canid groups analyzed from Předmostí^26^. As Bocherens and colleagues^26^ showed**,** the ‘Pleistocene wolf’ group had free access to all dietary resources and consumed mostly megafauna, while the ‘Palaeolithic dog’ group consumed primarily reindeer^26,27^. We observed a dietary distribution in the Magdalenian isospace within niches A and B, similar to the Předmostí canid groups^16^. Ultimately, the ecomorph hypothesis becomes a reasonable explanation for the observed genetic and isotopic patterns, but it fails to answer the question of why the Gnirshöhle canids would form a separate ecomorph while a high *δ*^15^N protein diet source was likely available nearby.

Besides environmental changes, humans can be a major driving factor to create ecological niches, and thus, domestication can lead to an ecomorph shaped by them^54,55^. Direct dating of the Gnirshöhle samples implied that canids could have lived in close vicinity of Magdalenian people, occupying the Hegau Jura, and subsequently adapted to a restricted diet, possibly under human influence. Thus, we consider the Gnirshöhle canids to likely represent an early phase in wolf domestication—facilitated by humans actively providing a food resource for those early domesticates (niche B^16^). Moreover, the high mitochondrial genome diversity could be explained by the fact that Magdalenian people would have arbitrarily drawn individuals from a large pool of canid genetic variation in the region some 15,000 years ago. We suggest that the proximity Magdalenian hunter-gatherers is the most parsimonious explanation for the genetic, isotopic, and archaeozoological patterns observed in the Gnirshöhle canids.

In conclusion, future investigations should include the analyses performed in our study, in addition to others for more detailed diet reconstructions (i.e. microwear analysis, amino acid isotope analysis), identification of the specimens’ geographic origin or range (i.e., sulfur, lead and strontium isotope analysis), as well as including nuclear genomes to further decipher details of the wolf’s domestication history. Intriguingly, a recent study focusing on the analysis of nuclear genomes of various ancient dogs suggested a single origin of modern dogs, but it failed to provide a geographic location for such an event^46^. While we cannot address the question of the domestication event’s singularity, our results support the hypothesis that the Hegau Jura was a potential center of early European wolf domestication. Such a scenario becomes plausible considering a close proximity of canids and humans thereby introducing a controlled, or at least a restrictive diet. This would foster their differentiation from wild conspecifics and may thus constitute a driving factor in the process of domesticating wolves. Lastly, we reiterated the importance of multidisciplinary approaches to investigate the origin of modern dogs, serving as a model for similar studies in the future.

## Material and methods

### Archaeological context and sample information

The Gnirshöhle (GN) is a small cave with two chambers (GN I and GN II) situated in the Bruder Valley close to Engen in the Hegau Jura of southwestern Germany. The new dates of the Gnirshöhle canid remains range from 15.5 to 15 ka cal BP (Figure S1, Table S1, Supplementary Note 3). Nearby are two other Magdalenian sites, Petersfels^56–58^, and Drexlerloch^59^. All three sites contain faunal remains dominated by reindeer and horses; in addition, Gnirshöhle provides a considerable number of canids^34^ (Supplementary Note 1). These remains are well-suited to explore early interactions between humans and canids. To shed light on these scenarios, we combined diverse datasets generated from various disciplines of archaeological sciences (archaeozoology, stable isotope analyses, and paleogenetics).

The archaeozoological investigations included 65 canid remains from Gnirshöhle (GN I: n = 60, GN II: n = 5) of which only two specimens were measurable, namely a mandible (GN-999, Figure S2, Tables S2 and S3), and a maxilla (GN-192, Figure S3, Tables S2 and S3). Well-preserved samples (larger than 0.5 cm, Figure S4) were selected for isotopic and genetic analysis. New isotopic data (*δ*^13^C, *δ*^15^N) were obtained for six out of eight canid remains of Gnirshöhle, one red fox from Bockstein Cave (BS-21), and one pre-LGM wolf from Hohle Fels (HF-1712) in the Swabian Jura (Supplementary Note 1, Table S1). Additionally, for the isotopic analysis, we included previously published isotope values from the specimens from Hohle Fels and Kesslerloch, which were genetically analyzed in this study. The palaeogenetic analyses focused on the mitochondrial genomes of canid samples from Gnirshöhle (n = 8, Fig. 3). We further generated mitochondrial genomes from additional canid remains (Supplementary Note 1), namely Hohle Fels (Swabian Jura, n = 11) including samples from the Aurignacian to the Late Paleolithic (Fig. 1): Umingmak (n = 5), a Palaeoeskimo site in northwestern Canada, Le Parc du Château (Auneau, n = 2), a Mesolithic site in France, and Frankfurt a. M. (Germany, n = 3), assigned either to Roman (n = 1) or Medieval periods (n = 2) and combined those with publicly available canid mitochondrial genomes (Table S9), including the specimens from Bonn-Oberkassel and Kesslerloch^25,41^. Although some of the analyzed samples come from outside of the region and period central to this manuscript, we included them in this paper to get as high as available to us diversity covered in our phylogenetic inference. Since canid mitochondrial lineages are widely geographically and temporally spread, the inclusion of North American wolves allowed us additionally to confirm the genetic continuity within the region.

### Archaeozoology: morphological and metrical methods

Archaeozoological identification of species and morphological classification of canid remains were done by taking measurements after von den Driesch^36^ and comparing them with the archaeozoological reference collection housed at the University of Tübingen, Germany. If fragmented specimens did not allow measurement, the size of the canid remains were compared to wolf or dog specimens from the reference collection and assigned as being either wolf-like, dog-like or *Canis* sp.

For metrical analysis of the GN-999 mandible, we focused on two measurements: the length of the tooth row (ALP_1_M_3_) and the maximal length of the first molar (M_1_) (CLM_1_ from mesial to distal). To enable a two-dimensional classification, we plotted the two measurements against each other and compared them with published metrical data from Germonpré and colleagues^35^ (‘Paleolithic dogs’, n = 31, ALP_1_M_3_ = 94.9 ± 3.5 mm, CLM_1_ = 28.9 ± 1.5 mm; ‘Pleistocene wolves’, n = 36, ALP_1_M_3_ = 101.2 ± 2.9 mm, CLM_1_ = 29.9 ± 1.3 mm; ‘Archaic dogs’, n = 27, ALP_1_M_3_ = 75.9 ± 3.6 mm, CLM_1_ = 22.5 ± 1.2 mm; modern ‘Northern wolves’, n = 35, ALP_1_M_3_ = 99.3 ± 3.8 mm, CLM_1_ = 29.5 ± 1.6 mm), and Pleistocene wolves (Nobis^60^, n = 1; Boessneck and von den Driesch^61^, n = 1; Napierala^33^, n = 9; Napierala and Uerpmann^32^, n = 1; Münzel^62^, n = 1, Modern wolves^60^, n = 4), post-LGM dogs^60^, n = 4) in addition to metric data from modern wolves (n = 3) from the archaeozoological reference collection mentioned above (Table S4). Other standardized measurements for GN-999 and the maxilla GN-192 after Von den Driesch^36^ are given in Supplementary Note 2.

### Stable isotopes: elemental and isotopic analysis

We performed new isotopic analyses from bone collagen for seven samples of canids from Gnirshöhle (*Canis* sp., n = 6), Hohle Fels (*Canis lupus*, n = 1) and Bockstein (*Vulpes vulpes*, n = 1). Collagen extraction followed the protocol outlined by Bocherens and colleagues^48^ and is further detailed explained in Supplementary Note 4. The process was performed in the Biogeology working group laboratory at the University of Tübingen (Germany). Isotopic measurements of collagen were undertaken in duplicate at the Institute of Environmental Science and Technology of the Universitat Autònoma de Barcelona (ICTA-UAB) using a Thermo Flash 1112 (Thermo ScientificVC) elemental analyzer coupled to a Thermo Delta V Advantage mass spectrometer with a Conflo III interface. All details for elemental and isotopic analysis are provided in Supplementary Note 4.

The isotopic ratios are expressed using the *δ* (delta) value as follows:

$$\delta ^{{13}} {\text{C }} = \left[ {{{\left( {^{{13}} {\text{C}}/^{{12}} {\text{C}}} \right)_{{{\text{sample}}}} } \mathord{\left/ {\vphantom {{\left( {^{{13}} {\text{C}}/^{{12}} {\text{C}}} \right)_{{{\text{sample}}}} } {\left( {^{{13}} {\text{C}}/^{{12}} {\text{C}}} \right)_{{{\text{reference}}}} - 1}}} \right. \kern-\nulldelimiterspace} {\left( {^{{13}} {\text{C}}/^{{12}} {\text{C}}} \right)_{{{\text{reference}}}} - 1}}} \right] \times 1000{\mkern 1mu} \left( {\textperthousand} \right)$$

$$\delta^{{{15}}} {\text{N }} = \, \left[ {\left( {^{{{15}}} {\text{N}}/^{{{14}}} {\text{N}}} \right)_{{{\text{sample}}}} /\left( {^{{{15}}} {\text{N}}/^{{{14}}} {\text{N}}} \right)_{{{\text{reference}}}} - { 1}} \right]{\text{ x }}1000 \, \left( \permil \right)$$

### Stable isotopes: niche modeling and dietary reconstruction

To reconstruct the niches of the sampled canids, we included newly analyzed *δ*^13^C and *δ*^15^N values of six canids from Gnirshöhle, one newly analyzed red fox from Bockstein, and published isotopic values of 14 canids^16,40^, and applied a multivariate cluster analysis (using JMP 14) to the *δ*^13^C and *δ*^15^N isotopic values of all canids. We then used R^63^ and the R package SIBER to calibrate the core niches (standard ellipse area = SEA) out of the clusters^64^. The core niche depicts the center of a niche that is calculated by using a Bayesian most-likelihood estimation and explains roughly 40% of the expected variability^64^. This method is more reliable for analyzing small sample sizes and recommended for niche interpretations by Jackson and colleagues^64^. For dietary reconstructions, we utilized the same prey groups that were defined by Baumann and colleagues^16^: ‘Megaherbivores’, ‘Ungulates’, and ‘Small mammals’ that were constructed from a database of 91 carbon and nitrogen stable isotopic values from herbivores (Table S5). To reconstruct the proportions of different prey groups in the protein fraction of the canids’ diet, we used MixSIAR^65^. More detailed information is presented in the Supplementary Note 4.

### Palaeogenetics: ancient DNA laboratory workflow and sequence analysis

All pre-amplification steps, i.e. the DNA extraction and library preparation, were performed in clean room facilities at the University of Tübingen fulfilling all requirements for ancient DNA work^66,67^. Amplification and mitochondrial canid DNA enrichment steps were performed in a separate laboratory also located at the University of Tübingen. Lastly, DNA libraries were either sequenced at the Max-Planck-Institute for Science of Human History in Jena, Germany, or at the Functional Genomic Center Zürich, Zürich, Switzerland. Detailed information can be found in Supplementary Note 5.

We used the Efficient Ancient Genome Reconstruction (EAGER) pipeline^68^, version 1.92.37 for bioinformatic analysis of the sequencing data (Supplementary Note 5). The quality of the sequencing reads was estimated by FastQC tool^69^ and the adapters were trimmed by AdapterRemoval^70^ version 2.2.1a, both tools are integrated in the EAGER pipeline. MarkDuplicates v2.15.0 (Picard Tools) was applied to remove duplicates for genome reconstruction^68^. To demonstrate the authenticity of ancient DNA reads, the program MapDamage^71^ is utilized by the EAGER pipeline to estimate the distribution and frequency of any misincorporations at the 3′- and 5′-ends of the DNA reads. Potential contaminated DNA reads in sequencing data for the individuals GN-14 and GN-106 were identified by a low frequency of misincorporations estimated by MapDamage^71^. To overcome potentially false reconstruction of mitochondrial genomes due to contamination, we applied PMD-tools^72^ to separate endogenous ancient DNA reads from modern contaminant reads with a threshold PMD score of three. For these two specimens, filtered reads were then used for mitochondrial genome reconstruction.

### Paleogenetics: phylogenetic analysis and timescale estimation

We used canid mitochondrial genome data published in previous studies^24,25,41,43,73^ to reconstruct a comprehensive phylogeny. In total, a multiple sequence alignment database of 244 genomes was constructed (MAFFT^74^), including 221 mitochondrial genomes recruited for our comparative dataset^24,25,41,43,73^ and the reconstructed genomes of our study. Before the phylogenetic tree reconstruction, we defined the best-fit model for our data^75^, using IQ-TREE^76^, to obtain the highest statistically supported phylogenetic tree. Subsequently, a phylogenetic tree was generated employing IQ-TREE^76^ using a maximum likelihood approach with an estimation of 10,000 bootstrap replications.

In order to estimate time-aware phylogeny in coalescent framework, we excluded the coyote mitogenomes, as well as three modern Tibetan/Mongolian wolves from China (NC011218, EU442884 and FJ032363) and one ancient Siberian wolf (MK936996) that in preliminary runs revealed an unexpectedly old divergence time, which could be attributed either to genetic isolation or comparatively stronger selective forces. The alignment of 236 mitogenomes was partitioned using PartitionFinder^77^ 2.1.1 into four partitions: (1) protein coding, gene position one (3782 nt length, TRN + I substitution model), (2) position two (3780 nt, TRN + G), (3) position three with rRNA and tRNA (7832 nt, TRN + I), and (4) non-coding (1050 nt, HKY + G). Bayesian phylogenetic and timescale estimation was performed using BEAST^78^ 1.10.4. Sample ages were used as tipdates for molecular clock calibration. Undated samples were assigned uniform age prior (0-100ky). Uncorrelated lognormal relaxed clock for each partition and Bayesian SkyGrid population model^79^ were used, as supported by the data. The MCMC chain was run for 200 M steps with sampling every 20,000th step. The first 20 M steps were removed as burn-in. Convergence and mixing were inspected (all ESS exceeded 100) and the SkyGrid plot was generated in Tracer^80^ v 1.7.1. Maximum Clade Credibility trees were generated in TreeAnnotator (part of the BEAST package) and visualized in FigTree^81^ 1.4.2.

### Palaeogenetics: estimation of genetic diversity

To investigate the genetic diversity in more detail, the mitochondrial genomes from Kesslerloch and Gnirshöhle were compared to modern dog and wolf mitochondrial DNA sequences^24,25,41,43,73^ assigned into four canid population groups: Kesslerloch (KSL), Gnirshöhle (GN), modern dogs, and modern wolves (Table S7 and S8). We calculated the nucleotide diversity for each predefined group via DnaSP^82^ v5 after excluding all sites with gaps for each group individually. The program estimated the nucleotide diversity Pi (π), sampling variance, and the standard deviation, defined by the square root of the variance^82^, according to well-established statistical algorithms^42,83^.

## Supplementary Information

Supplementary Information.

Supplementary Table S1.

Supplementary Table S9.

## Data Availability

The data will become available upon publication. The genetic data (raw sequencing data) can be found on NCBI (BioProject ID: PRJNA703747).
