# Supplementary material for: A refined proposal for the origin of dogs: the case study of Gnirshöhle, a Magdalenian cave site
Source: Sci Rep. 2021 Mar 4;11:5137. doi: 10.1038/s41598-021-83719-7 (PMC7933181; doi:10.1038/s41598-021-83719-7)
Supplement: Supplementary file 1 — Supplementary Information. [file 41598_2021_83719_MOESM1_ESM.pdf]

Supplementary Information

A refined proposal for the origin of dogs - The case study of Gnirshöhle, a Magdalenian cave site

Chris Baumann, Saskia Pfrengle, Susanne C. Münzel, Martyna Molak, Tatjana Feuerborn, Abagail Breidenstein, Ella Reiter, Gerd Albrecht, Claus-Joachim Kind, Christian Verjux, Charlotte Leduc, Nicholas J. Conard, Dorothée G. Drucker, Liane Giemsch, Olaf Thalmann, Hervé Bocherens, Verena J. Schuenemann

Supplementary Note 1: Archaeological background of the sites

Susanne C. Münzel, Christian Verjux, Charlotte Leduc, Liane Giemsch

Gnirshöhle and Bruder Valley (Engen, Hegau)

Gnirshöhle (GN) is a small cave with two chambers (GN I and II) situated in the Bruder Valley close to Engen (Hegau) and less than 30 km northeast of the site Kesslerloch in Thayngen (Canton Schaffhausen, Switzerland), well-known for the first mention of a dog by Rütimeyer[1] in 1875. Both caves are situated within the Hegau Jura (Figure 1). They first became accessible after the deglaciation of the westernmost region of Lake Constance[2] and both were occupied during the Magdalenian period by reindeer hunters[3]. Beside evidence of reindeer, the faunal remains of Kesslerloch included a considerable number of canid remains, also genetically investigated[4]. Near Gnirshöhle, the Magdalenian cave site of Petersfels, well-known for its venus-shaped personal ornaments made from gagate (fossil wood), is situated just a few hundred meters across the valley. The cave was mostly excavated in the 1930s by Eduard Peters[5,6] and later in the 1970s when Gerd Albrecht and his team from the University of Tübingen conducted excavations in front of the cave[7,8]. During this fieldwork, Gnirshöhle was discovered and excavated in three campaigns between 1977 and 1979[9]. Despite the restricted space, Gnirshöhle I and II yielded reindeer and horse bones with cut and impact marks as well as some cut marks on the remains of small game, including foxes, hares, and birds; but not on any canid bones. Additionally, signs of domestic activities were evidenced by the presence of worked organic tools, namely antler beams and typical Magdalenian bone needles[9]. Lastly, a third site in this area, named Drexlerloch, was discovered during construction work on a sewage water ditch in 1978. Only a few buckets of sediment were sampled which included some diagnostic lithics, ochre stained bones, and a few bones with cut marks, also dated to the Magdalenian period[10]. Seemingly, the Bruder Valley region was a hotspot of Magdalenian occupation dating to 16 - 15 ka cal BP[10]. Faunal remains of all three sites consisted mainly of reindeer and horse[8,10-12], while in Gnirshöhle canids were more commonly found. Sixty canid remains were identified in GN I and five in GN II, from a total of 4569 and 322 faunal remains, respectively[12]. Of special interest is a right mandible from Gnirshöhle I, which was found

superficially within the area excavated in 1977 and received no archaeological ID (herein referred to as GN-999). This mandible was proposed as a possible dog by Hans-Peter Uerpmann (pers. comm.), is relatively short (Figure 2 main text, Table S4) and exhibits tooth crowding between P_4 and M_1 (Figure S2). Given the recent radiocarbon dating (Figure S1, Supplementary Note 3) and that the cave only became accessible after the Last Glacial Maximum (LGM)[9], we presume that the canid remains were deposited during the time period when Magdalenian hunter-gatherers occupied the Bruder Valley. However, the question remains, whether or not the canid remains in Gnirshöhle are contemporaneous with the human occupation of this cave. Especially in Gnirshöhle I, the presumption of more carnivore activities was supported by a higher percentage of gnawed bones and the presence of coprolites. One coprolite was still intact and the others were documented as small concentrations of little bone splinters in the profile drawing[9]. Although coprolites have been predominantly associated with hyenas, which went extinct in the area before the LGM[13], research from North America proves that wolves' scats can also preserve as coprolites[14]. Thus, we conclude that there was canid activity in the chamber of Gnirshöhle I.

Hohle Fels cave in the Ach Valley (Schelklingen, Swabian Jura, SW-Germany)

Hohle Fels cave, situated east of Schelklingen/Ehingen, is one of six UNESCO World Heritage sites in the Swabian Jura close to Ulm[15]. The cave provides a stratigraphy covering the Middle Palaeolithic, Aurignacian, Gravettian, Magdalenian, and Late Palaeolithic. Thus, this site is an important place to study the cultural transition from Neanderthals to Anatomically Modern Humans[16-18]. The cave is one of the early archaeological discoveries of the 19th century. In 1870/71, the large cave hall was almost completely excavated by Oscar Fraas and Theodor Hartmann[19]. After short excavation episodes by Robert Rudolf Schmidt and Gustav Riek, the Institute for Urgeschichte of the University Tübingen excavated in the entrance tunnel from 1977-1979 and 1987-1996 under the direction of Joachim Hahn[20]. Since Hahn's passing, Nicholas Conard continues the research with annual excavations[19]. Of special importance, and part of the UNESCO World Heritage, are finds from the Aurignacian layers of the earliest mobile art, such as the Venus figurine and animal figurines made from mammoth ivory[21,22], personal ornaments[23,24], and the earliest musical instruments[25,26], all of which are attributed to Anatomically Modern Humans. The faunal composition during the pre-LGM of Hohle Fels is characterized by a Mammoth-steppe, including woolly mammoth and woolly rhino, wild horse, reindeer, ibex/chamois, hare, and cave bear for the herbivores, and brown bear, cave lion, hyena, wolf (subject of this study), red and arctic fox, and small mustelids for the carnivores. After the LGM, in particular during the Magdalenian, only wild horse, reindeer, and ibex/chamois remained in the Ach Valley as well as brown bear, wolf, and red and arctic fox[27-29] and cave lion in some places[30] . One archaeozoological highlight from Hohle Fels is the discovery of a cave bear vertebra with a lithic projectile lodged within the transversal process. To date, this is the first

secure proof of cave bear hunting and sheds light on hunting methods of cave bears during the Gravettian period[27].

Bockstein cave in the Lone Valley (Niederstotzingen, Swabian Jura, SW-Germany)

The Bockstein-complex in the Lone Valley is one of the six cave sites designated as UNESCO World Heritage sites[15]. Bockstein cave is a complex of eight different sites and one of the most important places for the Middle Palaeolithic sequence in southwestern Germany[31]. A correlation of the various excavations and their cultural chronology was evaluated by Petra Krönneck[32]. The fauna is dominated by wild horse, reindeer, and large bovids (*Bos /Bison*)[32,33]. The fox bone (BW-K 4.9.1934/21) sampled for the isotope analysis comes from Bockstein-Westloch, a small cave situated west of the large Bockstein cave. This particular part was excavated by Robert Wetzel in 1934[32]. The stratigraphy of Bockstein-Westloch revealed two Magdalenian and one Aurignacian layer. The fox sample originates from one of the Magdalenian layers (Table S1)[19].

Umingmak on Banks Island, N.W.T., Canada

Umingmak is a Palaeoeskimo site on Banks Island, the westernmost island of the Canadian Archipelago. The site was discovered 1965 by Taylor and McGhee[34], followed by excavations of the Institute for Early Prehistory and Quaternary Ecology (former Urgeschichte) (University Tübingen, Germany) directed by Hansjürgen Müller-Beck and his team for multiple seasons[35]: 1970, 1973, 1975, and 1987. From these excavations, research data, e.g., about lithics[36] or fauna[37], were published.

Umingmak is dated to between 3.6 - 3.4 ka cal BP and belongs to the Pre-Dorset culture[35,36,38,39]. The site is situated in the interior of Banks Island, close to Shoran Lake. In four campaigns, roughly 55 square meters in several different areas were excavated. The fauna in Umingmak (Inuit name for muskox) is dominated by muskox remains (>80%), some caribou, wolf, arctic fox, and arctic hare, as well as bird remains (mainly snow goose – *Anser caerulescens*) and some fish remains[37,40]. The hunting season of the Palaeoeskimos for muskox was early winter (November/December), while birds and fox were hunted during the summer, i.e. August[37,41]. During the field work in Umingmak, a human mandible was found close to the site[42]. During an isotopic investigation, the mandible was AMS radiocarbon dated to 4.3 ka cal BP[43], which is slightly older (but also Pre-Dorset period) due to improved dating methods than previous dates obtained for this site.

Le Parc du Château (Auneau), Mesolithic, France

The site of ‘Parc du Château’ at Auneau (Eure-et-Loir, France) is situated in the center of the Paris Basin about 20 km east of Chartres city. The site is located on a gently sloped mound at the confluence of a small river and a temporary stream, in the northeast part of the limestone Beauce plateau at the southwest limit of Fontainebleau Stampian sand extension. Discovered in

1979, the site was excavated over ca. 30 years under the direction of Jean-Pierre Dubois, Alain Villes and, more recently, Christian Verjux. The conducted research unearthed remains of occupation from the Middle Neolithic[44], from ca. 6,200 to 5,400 years BP, as well as more than 70 dug structures from the Mesolithic, which had been excavated between 1990 and 2001, over an area of 200 square meters. These last features, dug in the Fontainebleau sands, were embedded in a brown sand level that yielded Neolithic artifacts, while no occupation level corresponding to the Mesolithic has been preserved. The primary function of about 20 dug structures could be defined based on their content and arrangement: 3 burials, several intentional deposits (aurochs' skulls, red deer antlers), post holes, and some fireplaces. Nearly half of the other pits were used as middens and contained domestic wastes (e.g. flint and sandstone artifacts, animal bones, heated stone). The deeper pits have been considered as potential storage places filled with nuts[45]. The lithic industry, especially flint arrow-heads, and the radiocarbon dating point to a repeated occupation from 11,500 to 7,500 BP, but most of the dug structures were dated to between 10,200 and 9,000 BP. A large majority of the dug structures have yielded faunal remains, with diversified taxonomic composition. The bone remains (more than 2,500 including splinters) are well preserved. The bone surfaces are generally not altered, with only a few showing root etching, testifying to local bioturbations, however still marginal and superficial. Most of the bones do not show the surface alteration expected from weathering exposure, suggestive of a rapid burial. Some dug structures have provided a single bone element and about thirty of them included small faunal assemblages with less than 50 remains. In contrast, two pits (32 and 34) differed from that pattern with a corpus of 500 and 1,000 remains, respectively[46,47]. Aurochs (*Bos primigenius*) and roe deer (*Capreolus capreolus*) are the prominent species represented, followed by red deer (*Cervus elaphus*) and wild boar (*Sus scrofa*). Carnivores are less frequent with the occurrence of fox (*Vulpes vulpes*), wolf (*Canis lupus*), marten (*Martes martes*), and wild cat (*Felis silvestris*). Some bird bones belonged to the Anatidae family. No fish bones have been found despite the presence of a river close to the site. In Pit 41, a portion of an axial skeleton of a canid, composed of eleven vertebrae in articulation, was found under a small sandstone slab. One cervical vertebra from a canid, with excellent preservation, has been found in Pit 57, under stones used for a post hole. Some bones of undetermined canids were also collected from Pit 34.

Frankfurt (Roman and Early Medieval)

A dog skull (inventory number α19496**)** comes from the Roman NIDA, modern day Frankfurt am Main - Heddernheim. The upper left M^1 was sampled from this particular dog remain. Contextually, the dog's skull was found in the filling of a cistern in area 39 at a depth of 155-355 cm, which was placed in the IIB - III period[48] and is dated to around the end of the 3rd century AD.

Within the Franconian burial ground in Frankfurt am Main - Nieder-Erlenbach, two dog

skeletons, 1986.03.001.001 and 1986.03.001.002 (NER 13/Grave 21), were retrieved from dog burials dating to the Merovingian period. A total of seven dog burials have been found within the burial ground[49]. Based on the finds and features, NER 13/Grave 21 is dated to the Young Merovingian period (JM) II, and possibly JM III[49,50], i.e. from 640-670 AD and 670/80-720 AD, respectively[51,52]. For sampling, an upper right M^1 was used from both dogs.

Supplementary Note 2: Morphological concerns of specimens from Gnirshöhle and Hohle Fels

Susanne C. Münzel

The morphological variability of canids is considerably large. Morphological traits and metrics alone are not the determining factors when considering the ongoing debate for the domestication process[53-57]. A general consensus is held concerning the shortening of the snout, while the teeth remain large, resulting in tooth crowding, and in a subsequent step, the size of the teeth is reduced. However, tooth crowding alone is not a criterion for domestication either, as it has also been observed in wolves[57,58]. Conversely this means conclusions from measurements of single teeth are not appropriate to classify wolf or dog.

Gnirshöhle Mandible GN-999

Thus, we combined the length of the toothrow (ALP_1M_3) with the length of the M_1 (CLM_1) to measure the relation between snout length and length of the lower carnassial (M_1) (Figure 2). Unfortunately, these two measurements for the mandible were only available for the Kesslerloch wolves (see Pleistocene wolves in Figure 2); the dog from Kesslerloch is represented by a maxilla. For the other dog from Bonn-Oberkassel, the length of the tooth row in the mandible is considerably shorter, because the length of the alveoli was measured from P_1 to M_2, although the alveolus of M_3 is visible[59]. Furthermore, this mandible bears some pathologies, such as an agenesis (pathologically not erupted) of P_2 and P_3 contributing to the shortening of the jaw[60]. Given this pathology, this dog mandible from Bonn-Oberkassel was not comparable with Gnirshöhle (GN-999).

Gnirshöhle Maxilla GN-192

The metrics of the right maxilla GN-192 includes just P^4 (CLP^4 = 26.2 mm, CWP^4 = 14.1 mm) and places this specimen into the range of Pleistocene and modern wolves[3,59,61,62] (n = 8, CLP^4 = 25.4 ± 1.6 mm, CWP^4 = 13.3 ± 1.8 mm, Tables S2 and S3).

Hohle Fels Maxilla HF-530

When Edgard Camerós[63] brought the wolf maxilla (HF-530) from Hohle Fels from the uppermost Gravettian layer (AH IIB) as a possible domesticate into discussion, he triggered an avalanche of debate about 'Palaeo-dogs' in the pre-LGM of the Swabian Jura[60,64]. Camerós' argument for the maxilla HF-530 as a possible domesticate was the prominent protocon on the upper P^4 resembling that of the dog maxilla from Kesslerloch[63], but the debate about morphological traits and the discussion about teeth and mandible sizes in recent years demonstrates that with single traits and morphometrics alone, the classification is not possible if a wolf is domesticated or

not[53-57]. In the context of our research the maxilla HF-530 was recently dated to the Magdalenian (Supplementary Note 3).

However, all measurements of the maxilla HF-530 (P^4: CLP^4 = 26.1 mm, CWP^4 = 15.2 mm; M^1: CLM^1 = 21.5 mm, CWM^1 = 15.8 mm) were within the range of wolves.

Supplementary Note 3: Radiocarbon dating and other chronological concerns

Chris Baumann, Susanne C. Münzel

Gnirshöhle

Faunal remains of Gnirshöhle had been previously dated in the 1970s; however, at that time, large sample quantities of bone were required for one radiocarbon measurement, e.g. 90g, including bones of different species, such as five reindeer, one horse and one canid bone fragment. The old dates of primarily game species range between 12-13 ka BP (15-16.5 ka cal BP)[10]. Since the old dates have a large standard deviation and were a mixture of bones from different species, it was especially important to obtain new dates for the canids.

We used five collagen samples from canids for the new radiocarbon dating, all of which were also extracted for isotopic and genetic analysis (Table S1). Preparation for the radiocarbon dating followed the protocols of Hajdas and colleagues[65,66] and dating was performed at the Laboratory of Ion Beam Physics (ETH Zurich) by measuring the $^{14}C/^{12}C$ ratio using the MICADAS accelerator mass spectrometry (AMS). Finally, the ^{14}C dates were calibrated by using OxCal[67-69] v4.3.2. First, the mandible GN-999 was dated because the contemporaneity with the Magdalenian occupation was not clear due to its archaeological context (i.e. being found at the top layers) and the fresh appearance of the bone. Next, four other canid bones were dated, two from Gnirshöhle I (one right (GN-14) and one left (GN-106) tibia shaft fragment), and one atlas (GN-133) and one maxilla (GN-192) from Gnirshöhle II (Table S1). One important outcome of the radiocarbon dating is that both chambers of the cave, Gnirshöhle I and II, are contemporary and thus, the canid remains were deposited during the time range of Magdalenian occupation in the Bruder Valley between 16 and 15 ka cal BP[10] (Figure S1 and Table S1). This period still belongs into the Pleniglacial stage GS-2a (NGRIP-oxygen isotopes). The previously obtained dates seem to be slightly older, but the standard deviation, +/-300 years, is much larger than for the new dates[10]. The newly dated canids from Gnirshöhle are ca. 1,000 to 1,500 years older than the dogs from Kesslerloch or Bonn-Oberkassel (Figure S1).

Hohle Fels

Six wolf remains from Hohle Fels were dated, which were essential for the genetic diversity calculations. Two of the specimens (HF-530 and HF-1250.2) date to the Magdalenian and Late Palaeolithic periods, respectively (Table S1 and Figure S1). The dates of the other four canids (HF-912, HF-1965, HF-1035, and HF-1390, Table S1) align with the Gravettian and Aurignacian periods in Hohle Fels.

Some concerns with regard to the chronology in Hohle Fels

The maxilla HF-530 originates from the uppermost Gravettian layer (AH IIB), which is in direct contact to the Magdalenian layer. Therefore, pre- and post-LGM layers are sometimes difficult to distinguish in Hohle Fels. The assignment of a possible domesticate by Camerós and colleagues[63] initiated a discussion about ‘Palaeo-dogs’ in the pre-LGM of the Swabian Jura. Later, Prassack and colleagues[64] listed this specimen as a Gravettian dog and in the study of Janssens and colleagues[60], it has even been cited as a domesticate from the Aurignacian. In the light of this discussion, the sample was dated as part of this study and is of Magdalenian provenance. However, we cannot confirm the previous assignment as a dog.

Another chronological problem occurred for sample HF-912, a tibia shaft fragment found in layer GH 3AD/ AH IIAD assigned to the Magdalenian. However, during the excavation an admixture of some older fauna was recognized by a darker patina of the bones, while the typical Magdalenian fauna show a fresher ‘yellow-’ or ‘honey-colored’ patina. Sample HF-912 has a darker patina and most likely belongs to the pre-LGM period[29], which was corroborated by radiocarbon dating (31.5 – 31.2 ka cal BP, Table S1). This is also supported by the BEAST analysis (Figure 4, Figure S6, Table S6).

The samples HF-1250.1 and HF-1250.2, a 2nd metacarpal and a carpal bone, respectively, originated from a collapsed profile in one of the uppermost layers. The carpal bone (HF-1250.2) was dated to 13.3 - 13.1 ka cal BP. From an archaeozoological perspective, the two elements probably belong to one individual. This assumption is supported by the identical mitochondrial DNA sequence as well as by isotopic analysis. Accordingly, both samples were treated as one individual for isospace, and especially for all genetic downstream analysis, the sequencing data of both samples were merged. Both bone fragments could be assigned to the Late Palaeolithic period (14.2 - 11.6 ka cal BP), which is slightly younger than the Magdalenian period[10,70-72] (ca. 17.5 - 14.2 ka cal BP).

Supplementary Note 4: Stable Isotopes

Chris Baumann

Detailed methods for elemental and isotopic analysis

For the isotopic analysis, we cut small samples (0.3 – 0.7 g) from the bones using a Saeshin Forte 200 alpha micro-circular saw. After successive cleaning in purified Millipore water and acetone, we manually ground the samples to a powder (grain size less than 0.7 mm). To evaluate if collagen was still present in the samples, we performed a CN elemental analysis of the bone samples following the protocol of Bocherens and colleagues[73]. This analysis was performed at the Hydrogeochemisty department (University of Tübingen) using the Vario EL elemental analyzer. Sulfanilic acid from Merck was used as the international standard.
Collagen extraction followed the protocol of Bocherens and colleagues[74], and was performed in the laboratory of the Biogeology working group (University of Tübingen). Depending on the nitrogen content ($\%N_{bone}$) of each sample, as measured by the CNS analysis, we used 120 mg (4.0 – 4.5$\%N_{bone}$) to 450 mg (0.4 – 1.0$\%N_{bone}$) of bone powder for extraction. The collagen extraction process included a step of soaking the bone powder in 0.125 M NaOH between the demineralization and solubilization steps to eliminate lipids and humic acids, followed by freeze drying.
The isotopic measurements of collagen were undertaken in duplicate at the Institute of Environmental Science and Technology of the Universitat Autònoma de Barcelona (ICTA-UAB) using a Thermo Flash 1112 (Thermo ScientificVC) elemental analyzer coupled to a Thermo Delta V Advantage mass spectrometer with a Conflo III interface.
The laboratory used the international laboratory standard IAEA 600 (caffeine) as well as two in-house reference materials (modern collagen of camel and elk). An analytical error below 0.2‰ (1σ) was determined for $\delta^{13}C$ and $\delta^{15}N$ in all the repeated analyses. The reproducibility error for the amounts of C and N was lower than 2%.
Following the protocols of DeNiro[75] and Ambrose[76], we only used collagen samples with a carbon-to-nitrogen-ratio ($C{:}N_{coll}$) between 2.9 and 3.6 and a nitrogen percentage higher than 5% for palaeoecological interpretations.

Detailed methods of niche modeling and dietary reconstruction

To reconstruct the niches of the canids, we first applied a multivariate cluster analysis to the $\delta^{13}C$ and $\delta^{15}N$ values in JMP 14. As a result, we obtained three different clusters that were not dependent on species determination; instead, they depended exclusively on the individual trophic source reflected by carbon and nitrogen isotopes. We then used SIBER (Stable Isotope Bayesian Ellipses in R) to calibrate the niches out of the clusters[77]. With this R package, it was possible to reconstruct the complete niches (= convex hull[78]) and the core niches (= standard ellipse area[77]) of the canid communities. The complete niche includes all individuals of a niche

and is quite sensitive to sample size. In contrast, the core niche depicts the center of a niche that is calculated using the maximum likelihood estimation and explains 40% of the variability[77]. According to Jackson and colleagues[77], this is more reliable for analyzing small sample sizes and recommended for niche interpretations. Since Magdalenian sites in Central Europe were ecologically similar to those in Southwestern Germany and Northern Switzerland[79], we included 49 published δ^{13}C and δ^{15}N values from bone collagen of large and small herbivores from other parts in Central Europe to obtain a comprehensive isotopic dataset (Table S5).

For dietary reconstructions, we used the same prey groups as in Baumann and colleagues[80]: 'Megaherbivores,' 'Ungulates,' and 'Small mammals.' The 'Megaherbivore' group includes only mammoth (*Mammuthus primigenius*, n = 3) isotopic values. The 'Ungulates' group includes isotopic values from elk (*Alces alces*, n = 2), bison (*Bison* sp., n = 3), red deer (*Cervus elaphus*, n = 5), woolly rhinoceros (*Coeolodonta antiquitatis*, n = 2), horse (*Equus ferus*, n = 10), giant deer (*Megaloceros giganteus*, n = 2), and reindeer (*Rangifer tarandus*, n = 44). Finally, the 'small mammals' group includes isotopic values from ground squirrel (*Spermophilus major*, n = 4) and hare (*Lepus* sp., n = 17). To reconstruct the proportions of different prey groups in the protein fraction of the canids diet, we used MixSIAR[81] (Bayesian Mixing Models in R). MixSIAR allowed us to reconstruct the carnivores' most likely diet based on the nitrogen and carbonate isotopes from their bone collagen relative to isotopic values from prey species. In order to use MixSIAR, it was essential to establish distinct prey sources as well as comparative Trophic Enrichment Factor (TEF) values. TEF values reflect the enrichment of nitrogen and carbon in collagen. The stable isotope composition of a predator differs from the composition of its prey predictably. The TEF values correspond to the difference between the stable isotope ratios of the consumer (predator collagen) and its diet (prey collagen) and are the result of the discrimination of stable isotopes due to the behavior and physiology of the consumer[82-84]. For this study, we used TEF values (Δ^{13}C = 1.1 ± 1.1‰; Δ^{15}N = 3.2 ± 1.8‰) from a study on modern fox by Krajcarz and colleagues[83].

To get a robust statistical analysis, we set the MCMC (Markov Chain Monte Carlo[81]) chain length to 1,000,000 with a burn-in of 500,000 in 3 chains. We verified the model convergence with Gelman-Rubin and Geweke tests, which shows model convergence, if the values are near 1, but in most analyses, values below 1.1 are acceptable[85]. Additionally, the Geweke test compares the mean of the first part of each chain with the second part's mean, using a two-sided z-test. If both means are the same, the model is convergent[81].

Results of the elemental and isotopic analysis, and convergence test for MixSIAR

The percentage of nitrogen in bone (%N_{bone}) was measured in eight samples. However, only six of them confirmed the favorable conditions of preservation (1.1 – 3.1% N_{bone}), establishing

quantitatively that collagen is well-preserved in the samples. Moreover, the atomic $C:N_{coll}$ ratios of all analyzed extracts (3.3 – 3.4) confirmed that collagen preservation was appropriate for the interpretation of the isotopic analysis (Table S1, Table S5). Among the isotopic values, we found a clear difference between the newly analyzed fox from Bockstein and canids from Gnirshöhle. The red fox from Bockstein has a $\delta^{13}C$ value of −20.3‰ and a $\delta^{15}N$ value of +5.6‰. The Gnirshöhle canids range in their $\delta^{13}C$ values between −19.9 and −19.2‰ and their $\delta^{15}N$ values between +5.3 to +6.1‰.

The MixSIAR[81] calculated model for dietary reconstructions showed convergence in both error tests. Both diagnostics tested 40 variables of the model. In the Gelman-Rubin test, none of these variables were higher than 1.01. Additionally, the Geweke diagnostic revealed no unequal variable in chains 1, 2, and 3 out of 40. Therefore, the calculated model is usable for dietary reconstruction.

Supplementary Note 5: Ancient DNA laboratory workflow, sequencing and DNA data processing

Saskia Pfrengle

Description of ancient DNA workflow

DNA extraction and pre-amplification steps of all analyzed canid samples from Hohle Fels and Gnirshöhle were undertaken in the clean room facilities at the University of Tübingen. Post-amplification steps were performed in a separate DNA laboratory at the University of Tübingen. All laboratories fulfill the requirements for ancient DNA research[86,87].

To minimize the risk of potential contamination with modern DNA, the surface of all bone and tooth samples were initially UV irradiated from all sides at least for 30 minutes. For DNA extractions, we applied a well-established guanidine-silica based extraction protocol developed for ancient DNA work[88]. The extracted DNA was converted into Illumina sequencing libraries[89] using 20 µl of each DNA extract. In a further step, dual barcodes (indexes) were added to the prime ends of the DNA-libraries[90]. We prepared between one and four sequencing libraries per each sample. For extraction and library preparation, negative controls were carried in parallel throughout the entire laboratory procedure. The DNA concentration, defined by the copy number of DNA fragments in the sample, was estimated after each step of library preparation, by means of quantitative polymerase chain reaction (qPCR, Roche LightCycler) using conforming primers[89].

The indexed sequencing libraries were then amplified again with Herculase II Fusion using the following conditions: 1X Herculase II buffer, 0.4µM IS5 and 0.4µM IS6 primer[89], Herculase II Fusion DNA polymerase (Agilent Technologies), 0.25 mM dNTPs (100 mM; 25 mM each dNTP), and 0.5 - 4 µl barcoded library as template in a total reaction volume of 100 µl. The amplification thermal profile was executed as described: initial denaturation for 2 min at 95°C, denaturation for 30 sec at 95 °C, 30 sec annealing at 60 °C, 30 sec elongation at 72 °C for three to 20 cycles following by a final elongation step for 5 min at 72 °C. Afterwards, the amplified DNA was purified by a MinElute purification step and DNA was eluted in 20 µl TET. We measured the concentration of the amplified sequencing libraries using Bioanalyzer (Agilent Technologies) and a DNA1000 lab chip from Agilent Technologies.

For the enrichment of the canid mitochondrial DNA (mtDNA), four to six canid DNA libraries were pooled to reach a total of 2,000 ng of DNA, used for the experimental procedure. We performed the enrichment of the canid mitochondrial DNA following bead capture protocols as described by

Maricic and colleagues[91], and Furtwängler and colleagues[92], previously shown to be successful in studies of the mitochondrial genome of canids[4,93].

DNA sequencing

The enriched libraries were sequenced at an Illumina HiSeq4000 platform at the Max-Planck-Institute for Science of Human History in Jena, or either on a HighSeq4000 platform or a NextSeq500 platform at the Functional Genomic Center Zurich in Zurich using standard protocol and chemicals for Illumina sequencing. Libraries were sequenced using 75+8+8 cycles (single-end reads) at Max-Planck-Institute for Science of Human History in Jena and using 2*75+8+8 cycles (paired-end reads) at the Functional Genomic Center Zurich.

DNA sequencing reads processing

For post-sequencing processing, we used the bioinformatics tool Efficient Ancient Genome Reconstruction (EAGER) pipeline[94] version 1.92.37, a tool specifically developed for ancient DNA sequencing data analysis. The quality of the sequencing data was estimated by FastQC tool[95] and the sequencing reads were adapter trimmed with AdapterRemoval[96] ver. 2.2.1a. For the mapping, duplicate reads were removed using MarkDuplicates[97] v2.15.0. Read mapping and alignment was performed by CircularMapper[94] version 1.0 using the publicly available mitochondrial dog genome[98] (NC_002008.4) as reference. To demonstrate the authenticity of ancient DNA reads, the program MapDamage[99] is utilized by the EAGER pipeline to estimate the distribution and frequency of any misincorporations at the prime-ends of the DNA reads, which accumulate due to degradation processes of the DNA over the time. For the reconstruction of canid mitochondrial genome, the read mapping was performed using of following parameters: BWA seedlength (-l) of 1,000 to effectively turn off seeding, BWA Max # Diff (-n) of 0.01 allowing less differences of reads to the reference sequence, and BWA Qualityfilter of 20, to discard reads with a mapping quality score lower than 20. After read mapping, the genome coverage was estimated by QualiMap[100] integrated within the EAGER pipeline. First, a full genotyping was performed using GATK[101] and then the genome was reconstructed by VCF2Genome[94] with the following parameters: the minimal genome quality (GATK) was set at 20 and the minimum SNP allele frequency was set at 0.75.

Post-mortem degradation (PMD) of DNA is used as an authentication criteria of endogenous ancient DNA[99]. Potential contaminated DNA reads in sequencing data for the individuals GN-14 and GN-106 were identified by a low frequency of misincorporations estimated by MapDamage[99], compared to the estimated frequency of misincorporations for sequencing reads of individuals from the same archaeological site and time. To overcome potentially false reconstruction of mitochondrial genomes due to contamination, we applied PMD-tools[102] to separate endogenous ancient DNA reads from modern contaminant reads with a threshold PMD

score of three. Filtered reads were then used for genotyping by GATK[101] and genomes were reconstructed by VCF2 Genome[94] using the same parameters as described above.

Supplementary Note 6: Phylogeny of the non-Magdalenian canids

Saskia Pfrengle

Phylogenetic Results

The mitochondrial genomes reconstructed from the specimens from Hohle Fels (HF-1965, HF-1035, HF-1174, HF-1553, HF-1712, HF-912, and HF-1390) were also positioned in the cluster of pre-LGM canids (Figure S5). The two closely related Mesolithic canid mitochondrial DNA sequences originating from Auneau (APC-19 and APC-20) clustered with two ancient wolf genomes (Figure S5)[4,93], one from Switzerland (KSL-60), and one from Belgium (BEL-2506.1). Additionally, the Roman specimen (F-α19496) was positioned basally to the two modern mitochondrial DNA sequences of the dog D clade (Figure S5). Taken together, these three ancient mitochondrial DNA sequences, APC-19, APC20, and F-α19496, positioned close to the modern dog haplogroup D specimens, were part of a sub-branch encompassing 15 specimens ranging in the time from the Magdalenian period to modern times. Moreover, we observed that the Medieval dog specimens from Frankfurt, were placed within the dog A clade.

In the phylogenetic tree, the mitochondrial genome of HF-1250 is closely located to the Magdalenian mitochondrial genome of the GN-999 specimen (Figure S5). The four Canadian mitochondrial genomes clustered together and were positioned closely and basally to a cluster of modern wolf mitochondrial DNA sequences originating from North America (Figure S5).

Discussion of the Phylogenetic Results of the non-Magdalenian canids

While the main focus of this study concentrates on the interpretation of the new southwestern German mitochondrial genomes, it was also necessary to assemble a more comprehensive dataset with which those samples could be more precisely analyzed phylogenetically. This included the addition of canid mitochondrial genomes from varying archaeological periods and origins. The placement of the Canadian mitochondrial DNA sequences basally to a group of 21 North American modern wolves indicated genetic continuity of the maternal lineage over the last 2,300 years in North American wolf populations. The placement of the two Merowingan canids' mitochondrial genomes suggested that the modern dog haplogroup A was represented in German dogs since the medieval time period. The Mesolithic specimens APC-19 and APC-20 clustered with KSL-60 and BEL-2506. These four DNA sequences are placed basal to the modern haplogroup D dogs, thus we suggest that these canids represent a sister clade of modern dog haplogroup D. Furthermore, the Roman mitochondrial genome was located in a

sister clade of the modern dog D, hence this canid was also identified as an ancient specimen representing a sister taxa of the modern haplogroup D.

Lastly, contrary to previous findings [93], and mainly due to the fact that we included dog mitochondrial genomes in our phylogenetic analyses, we were able to more precisely assign the two samples KSL-189 and Siberia5. With regard to the KSL-189, we can provide genetic evidence that this sample is more closely associated with dogs rather than with wolves , which matches previous morphological evidence[61]. The same holds true for Siberia5, a sample previously considered as a wolf but herein receiving genetic support to potentially resemble a dog.

Supplementary Figures

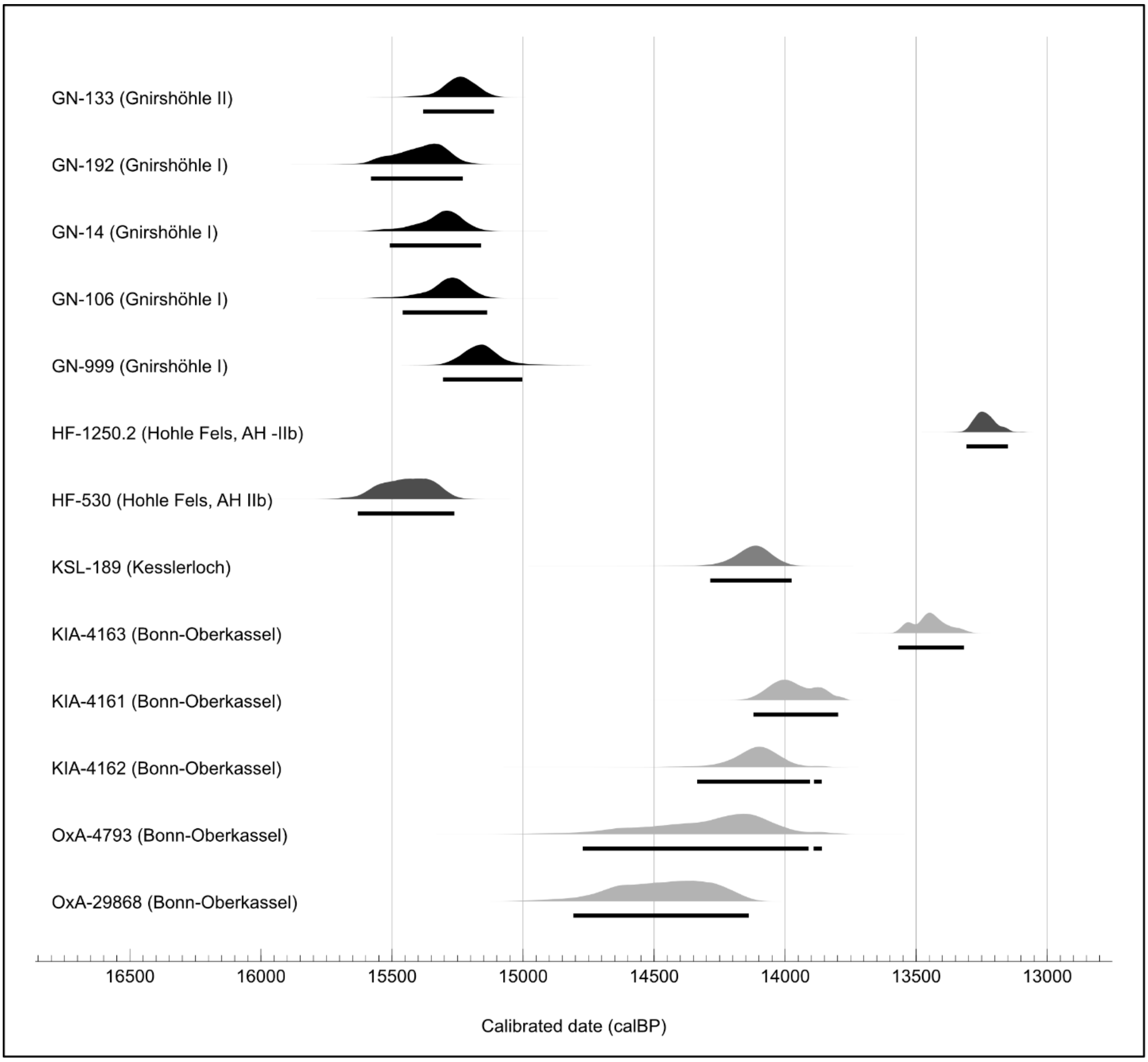

Figure S1: Radiocarbon dates of Magdalenian and Late Palaeolithic canids. Radiocarbon dates are given in cal BP.

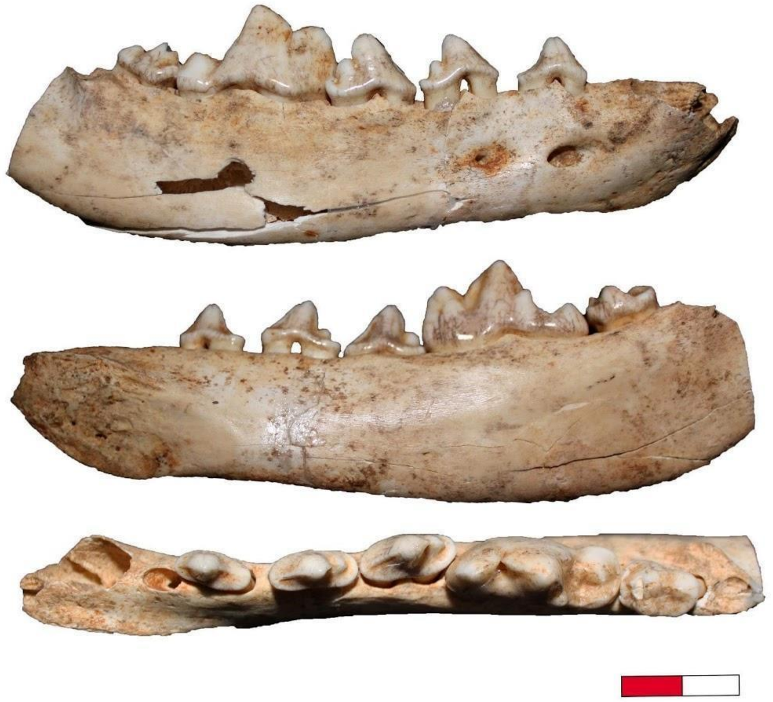

Figure S2: Image of the mandible GN-999 from Gnirshöhle I (Scale: 20mm).

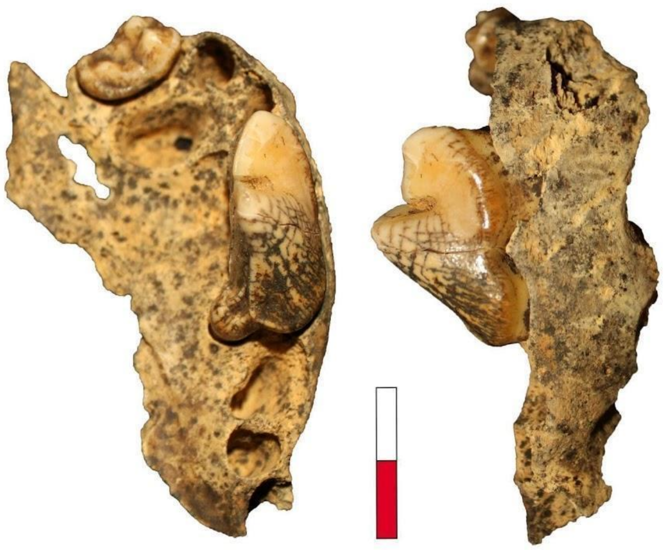

Figure S3: Image of the maxillary fragment GN-192 from Gnirshöhle II (Scale: 20mm)**.**

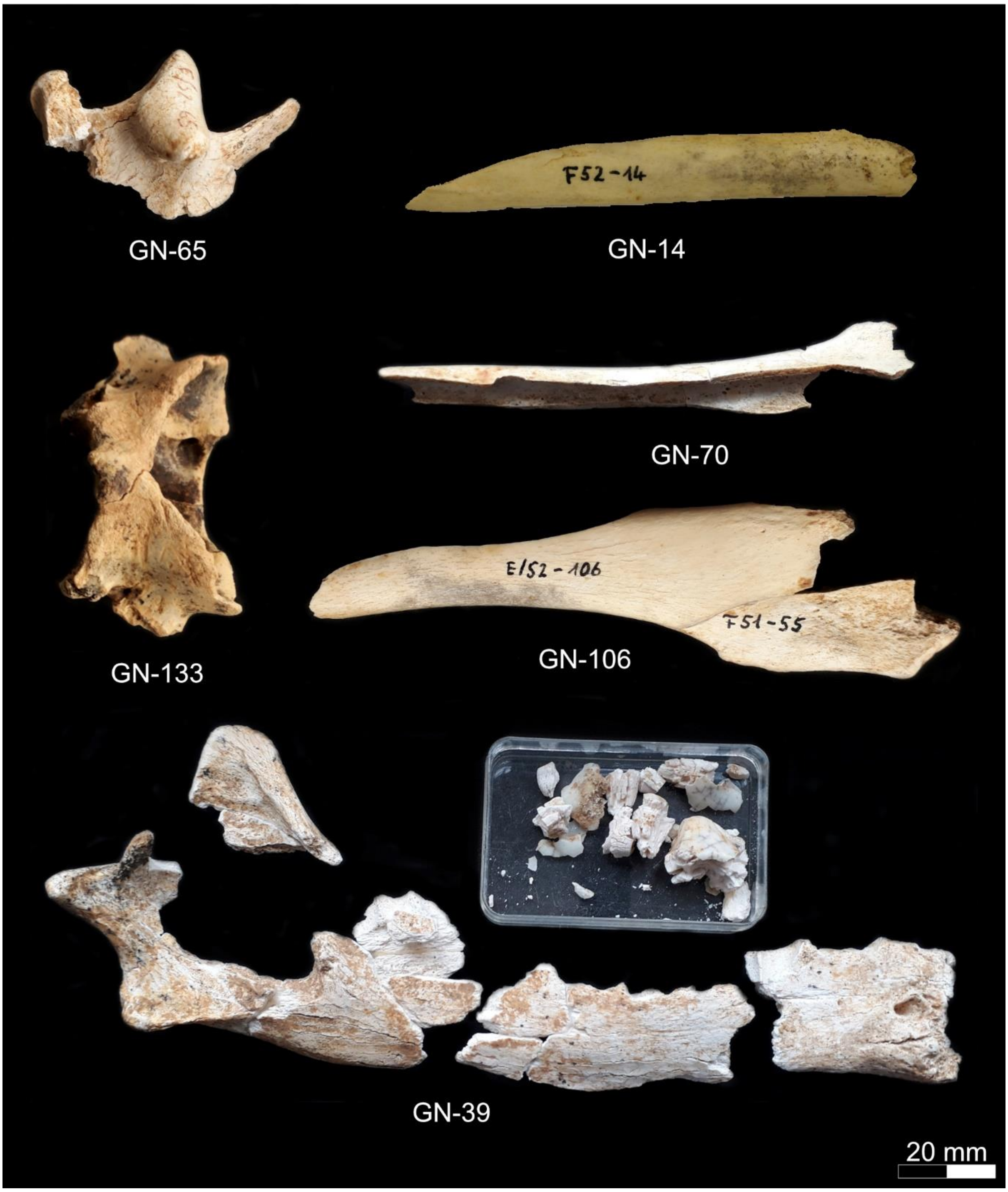

Figure S4: Images of all additional examined bone fragments from Gnirshöhle.

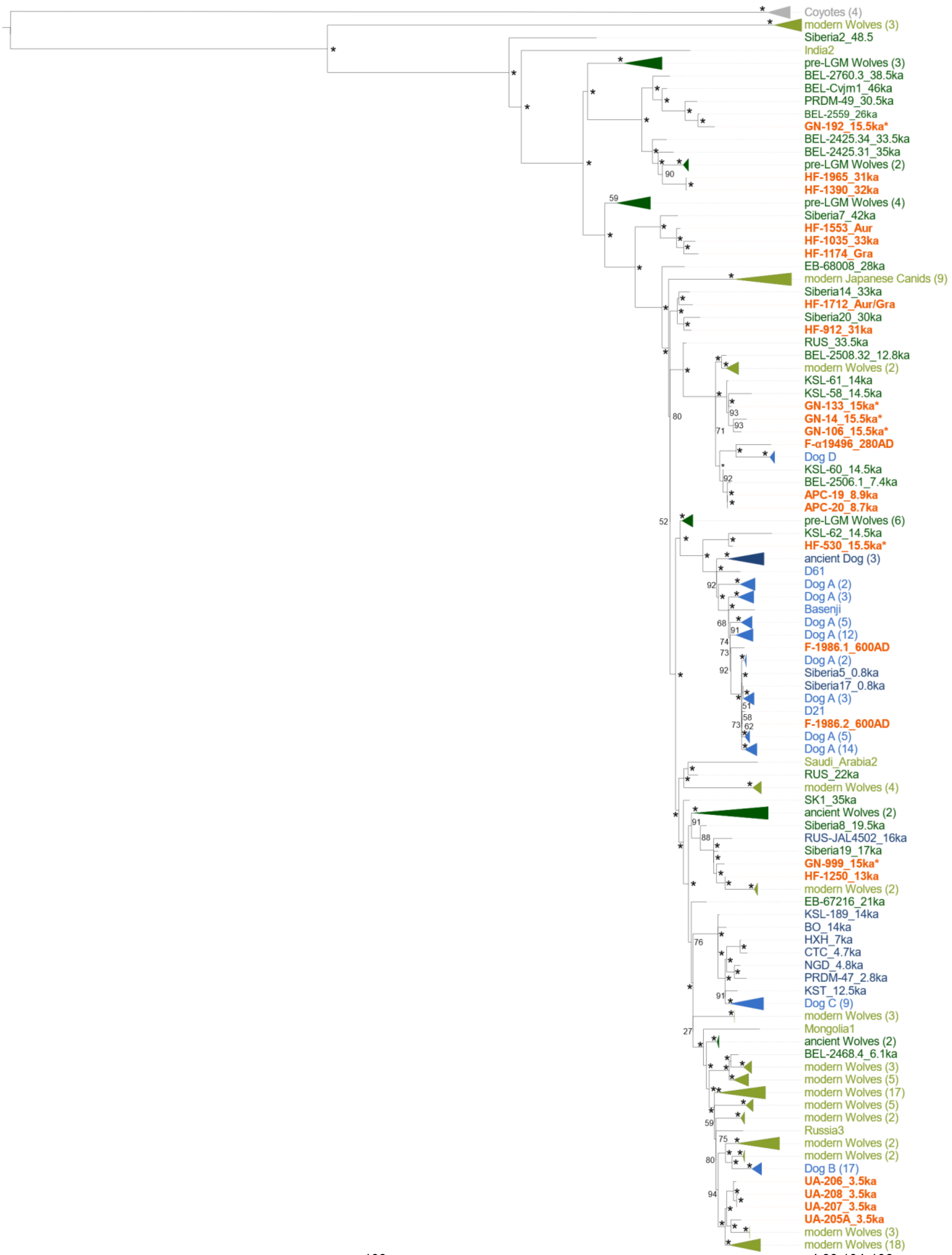

Figure S5: Maximum Likelihood tree[103] of the canid mitochondrial DNA sequences[4,93,104-106]. Tree was reconstructed by using 10,000 bootstrap replicates, bootstrap values above 95 are visualized at the node with an asterisk. Samples of our study are represented in orange and bold letters, in addition Magdalenian samples are marked with a star. Ancient Dogs are labeled in dark blue and Modern Dogs in light blue. Ancient Wolves in dark green and Modern Wolves in light green. The age of the samples is included in the sample name as an additional information, ages older than 2000 years are given in ka (cal BP), younger in AD, for undated samples the archaeological period is given.

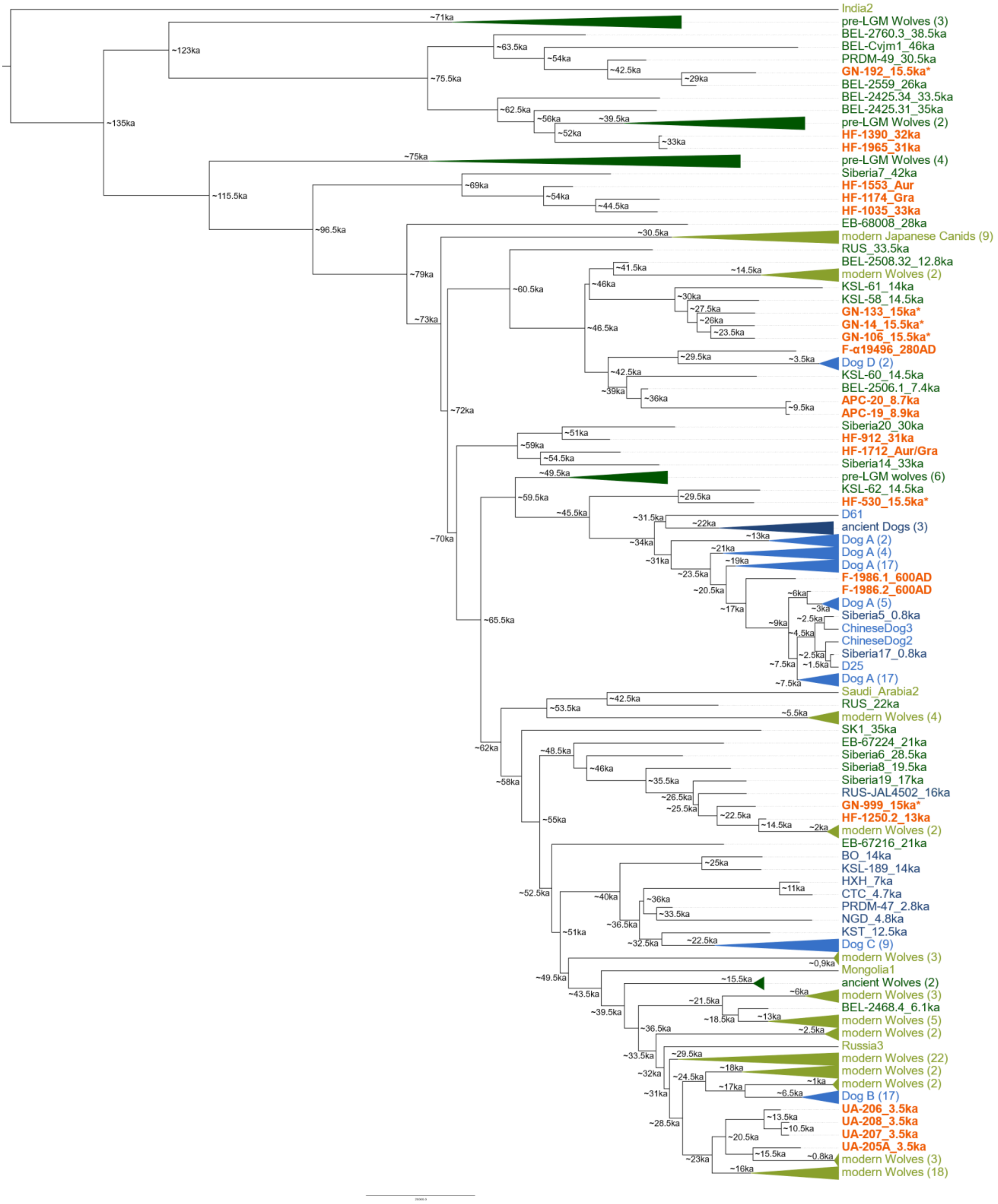

Figure S6: Bayesian Maximum Credibility tree estimated for 236 canid mitogenomes using BEAST[107] 1.10.4. Coyotes, three modern Tibetan/Mongolian wolves from China and one ancient Siberian wolf were excluded from the analysis (see Material and methods section of the main text). Sample ages were used as tipdates for molecular clock calibration. Undated samples were assigned each a uniform age prior (0-100ky). Color code and sample names are adopted from the Maximum Likelihood tree. Labels at nodes indicate respective estimated time to the MRCA.

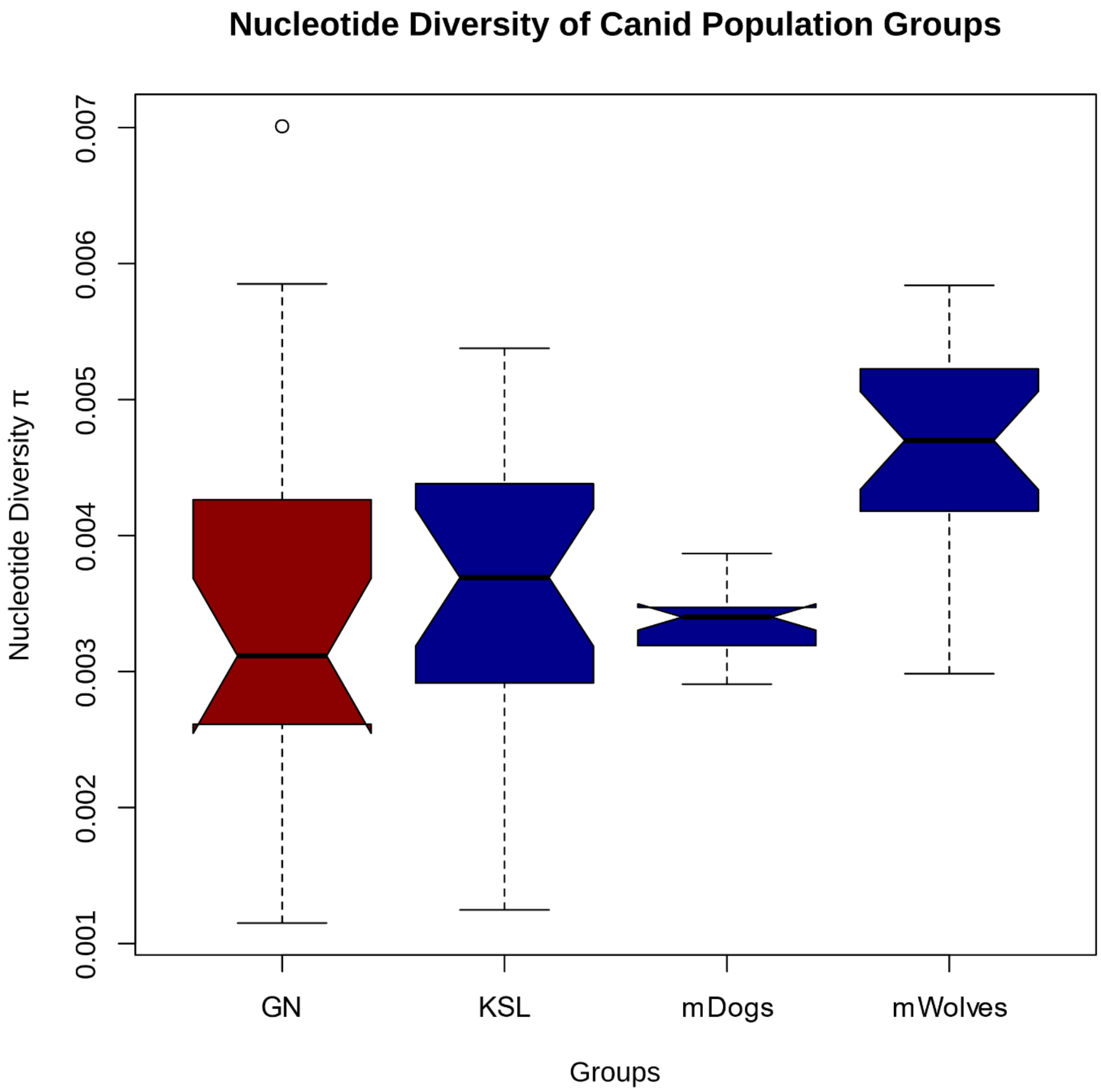

Figure S7: Visualization of Nucleotide Diversity π calculated for the Gnirshöhle canids (GN) (red boxplot), and for Kesslerloch canids (KSL), modern dogs (mDogs), and modern wolves (mWolves) (blue box plots).

Supplementary Tables

Table S1: Archaeological context of the canid samples in chronological order (GH=geological horizon; AH=archaeological horizon; (1) bone comes from a collapsed profile, after dating it was assigned to the Late Palaeolithic; (2) the maxilla is from the uppermost Gravettian layer AH IIb, which is in direct contact with the Magdalenian; the new date corrected its affiliation into the Magdalenian; (3) these Umingmak samples were not directly dated, the time range is given by earlier radiocarbon dates[35,38] of the archaeological layer. Furthermore, the table provides additional and detailed information about all the analyzed samples, regarding genetic and isotopic analyses. (4) Isotopic values are published by Baumann and colleagues[80], and (5) by Baumann and colleagues[108]

(Separate file)

Table S2: Greatest length (gL) and greatest breadth (gB) of teeth and alveoli (in mm).

	Single teeth and alveoli	gL [mm]	gB [mm]	gL-Alveolus [mm]	gB-Alveolus [mm]
GN-999	Caninus	not present	not present	13.60	10.88
	P1	not present	not present	6.20	4.06
	P2	11.86	5.36	12.53	5.00
	P3	13.64	5.82	14.62	5.68
	P4	15.26	6.90	15.14	7.60
	M1 (CLM1)	27.30	10.72	27.20	9.18
	M2	12.52	8.90	13.00	7.60
	M3	not present	not present	not measurable	not measurable
GN-192	Caninus	not present	not present	not present	not present
	P1	not present	not present	not present	not present
	P2	not present	not present	not present	not present
	P3	not present	not present	12.20	8.74
	P4	24.74	14.40	24.78	13.90
	M1	not present	not present	14.74	19.66
	M2	9.34	13.24	not measurable	not measurable

Table S3: Measurements at the mandible GN-999 and maxilla GN-192 (in mm).

Measurements at the mandible GN-999	[mm]	No. v.d. Driesch, p. 61
Length: aboral border of M3 alveolus to aboral border of C alveolus	97.50	7
Length of the cheek tooth row 1 (ALP1M3)	94.40	8
Length of the cheek tooth row 2 (ALP2M3)	88.44	9
Length of the molar row (LM)	46.95	10
Length of the premolar row P1-P4	48.72	11
Length of the premolar row P2-P4	46.64	12
Greatest thickness of the jaw below M1	12.57	17
Height of mandible behind M1 (Hm1)	26.58	19
Height of mandible between P2/P3 (Hp2p3)	21.88	20
**Measurements at the maxilla GN-192**	**[mm]**	**No. v.d. Driesch, p. 42**
Length of the molar row (LM1M2)	23.34	16

Table S4: Metric data of GN-999 taken after von den Driesch[109], compared to seven additional canid groups. The metrics are visualized in Figure 2.

Site	ID	CLP1M3 (mm)	CLM1 (mm)	Reference
Gnirshöhle I	GN-999	94.4	27.3	this publication
Pleistocene wolves (n=7)				
Brillenhöhle	AHIV (Magd)	104.0	31.0	Boessneck/ v.d.Driesch[110]
Kesslerloch	KSL KON1/616 (Magd)	100.2	32.5	Napierala[3]
Kesslerloch	KSL KON1/617 (Magd)	91.2	27.7	Napierala[3]
Kesslerloch	KSL M001/51 (Magd)	99.2	29.5	Napierala[3]
Kesslerloch	KSL M001/52 (Magd)	103.0	32.0	Napierala[3]
Kesslerloch	KSL N001/28 (Magd)	96.7	29.6	Napierala[3]
Geißenklösterle	GK 87/461 (Aurig)	94.5	29.7	Münzel[62]
Reference collection, modern wolves (n=4)				
Shoran Lake, Banks Island, Canada	CN3	98.6	29.3	
Shoran Lake, Banks Island, Canada	CN-E39	96.2	29.5	
Alaska, USA (male)	CN47	99.9	29.2	
ZFMK Bonn	78,268	97.0	32.6	
Reference collection, Uni Tübingen, modern dogs (n=2)				
Dog	CN119	83.7	24.8	
Inuit dog, Banks Island, Canada (P1 is missing, measurement estimated)	CN19	91.2*	24.4	
Canid groups according to Gemonpré and colleagues[55]				
Archaic dogs (n=27)		75.9 ± 3.6	22.5 ± 1.2	Gemonpré and colleagues[55]
Palaeolithic dogs (n=31)		94.9 ± 3.5	28.9 ± 1.5	Gemonpré and colleagues[55]
Northern wolves (n=35)		99.3 ± 3.8	29.5 ± 1.6	Gemonpré and colleagues[55]
Pleistocene wolves (n=36)		101.2 ± 2.9	29.9 ± 1.3	Gemonpré and colleagues[55]

Table S5: Isotopic data from the Magdalenian used for the estimation of isospace and dietary niche reconstruction. 'Dietary resource group' is only given for prey species.

Site	Taxon	ID in this study	Reference	Published LabID	C:N$_{coll}$	$\delta^{13}C_{coll}$ [‰]	$\delta^{15}N_{coll}$ [‰]	Dietary resource group
Kesslerloch	*Canis lupus*	KSL-58	Baumann et al.[80]	M 58	3.5	-20.1	7.1	
Kesslerloch	*Canis lupus*	KSL-62	Baumann et al.[80]	M 62	3.4	-20.0	7.9	
Schussenquelle	*Canis lupus*	SCH-11	Bocherens et al.[79]	SCH-11	4.2	-20.3	5.8	
Kesslerloch	*Canis lupus (familiaris)*	KSL-13	Bocherens et al.[79]	KSL-13	3.4	-19.0	5.7	
Kesslerloch	*Canis lupus (familiaris)*	KSL-14	Bocherens et al.[79]	KSL-14	3.4	-19.1	6.4	
Kesslerloch	*Canis lupus familiaris*	KSL-189	Baumann et al.[80]	KSL-44	3.3	-19.2	5.9	
Hohle Fels	*Vulpes lagopus*	HF-211	Baumann et al.[80]	VLP-8	3.3	-20.6	5.3	
Geißenklösterle	*Vulpes vulpes*	GK-17	Baumann et al.[80]	VLP-6	3.4	-20.6	5.1	
Gnirshöhle II	*Vulpes vulpes*	GN-171	Baumann et al.[80]	PLC-21	3.3	-20.6	4.5	
Kesslerloch	*Vulpes vulpes*	KSL-17	Bocherens et al.[79]	KSL-17	3.3	-19.6	4.7	
Vogelherd	*Vulpes vulpes*	VH-7185	Baumann et al.[80]	PLC-12	3.3	-19.7	8.2	
Schussenquelle	*Alces alces*		Bocherens et al.[79]	SCH-6	3.4	-20.2	3.0	Ungulates
Schussenquelle	*Alces alces*		Bocherens et al.[79]	SCH-7	3.4	-19.8	2.9	Ungulates
Bavans	*Cervus elaphus*		Bocherens et al.[79]	BVN-9(2)	3.2	-20.8	2.1	Ungulates
Rochedane	*Cervus elaphus*		Drucker et al.[111]	RCD500	3.2	-19.9	0.8	Ungulates
Rochedane	*Cervus elaphus*		Drucker et al.[111]	RCD10900	3.0	-20.4	2.0	Ungulates
Rochedane	*Cervus elaphus*		Drucker et al.[111]	RCD11000	3.2	-20.6	2.4	Ungulates
Grotte de Chaze II	*Coeolodonta antiquitatis*		Bocherens et al.[79]	ARL-1	3.1	-20.3	5.3	Ungulates
Champréveyres	*Equus ferus*		Bocherens et al.[79]	CHM-2	3.0	-19.8	1.6	Ungulates
Champréveyres	*Equus ferus*		Bocherens et al.[79]	CHM-4	3.0	-20.1	1.5	Ungulates
Monruz	*Equus ferus*		Bocherens et al.[79]	MRZ-1	3.1	-20.4	1.3	Ungulates
Monruz	*Equus ferus*		Bocherens et al.[79]	MRZ-2	3.2	-20.6	2.1	Ungulates
Hohlenstein Stadel	*Megaloceros giganteus*		Immel et al.[112]	HST-28	3.4	-20.7	2.2	Ungulates
Buttentalhöhle	*Rangifer tarandus*		Bocherens et al.[79]	TUB-57	3.3	-19.7	1.7	Ungulates
Fellställe	*Rangifer tarandus*		Drucker et al.[113]	FLS-7	3.3	-19.8	2.1	Ungulates
Felsställe	*Rangifer tarandus*		Drucker et al.[113]	FLS-8	3.3	-19.3	2.2	Ungulates
Felsställe	*Rangifer tarandus*		Drucker et al.[113]	FLS-9	3.3	-20.2	2.1	Ungulates

Grotte de la Baume Noire	*Rangifer tarandus*		Bocherens et al.[79]	FRT 1	3.3	-19.5	3.7	Ungulates
Grotte du Chaumois-Boivin	*Rangifer tarandus*		Bocherens et al.[79]	BLS 1	3.3	-19.5	2.4	Ungulates
Grotte Grappin	*Rangifer tarandus*		Bocherens et al.[79]	ARL 6	3.1	-19.1	2.8	Ungulates
Ranchot	*Rangifer tarandus*		Bocherens et al.[79]	RAN-10	3.0	-19.1	3.9	Ungulates
Rochedane (D1)	*Rangifer tarandus*		Bocherens et al.[79]	RCD100	3.2	-19.3	1.7	Ungulates
Rochedane (D1)	*Rangifer tarandus*		Bocherens et al.[79]	RCD200	3.2	-19.7	2.7	Ungulates
Rochedane (D1)	*Rangifer tarandus*		Bocherens et al.[79]	RCD400	3.2	-19.5	1.3	Ungulates
Rochedane (D1)	*Rangifer tarandus*		Bocherens et al.[79]	RCD10800	3.2	-20.3	2.1	Ungulates
Kesslerloch	*Bison sp.*		Bocherens et al.[79]	KSL-10	3.4	-19.8	2.2	Ungulates
Kesslerloch	*Bison sp.*		Bocherens et al.[79]	KSL-11	3.3	-20.0	2.4	Ungulates
Kesslerloch	*Bison sp.*		Bocherens et al.[79]	KSL-12	3.2	-20.3	2.3	Ungulates
Kesslerloch	*Cervus elaphus*		Bocherens et al.[79]	KSL-45	3.4	-20.2	2.0	Ungulates
Kesslerloch	*Coeolodonta antiquitatis*		Bocherens et al.[79]	KSL-7	3.3	-19.9	2.1	Ungulates
Kesslerloch	*Equus ferus*		Bocherens et al.[79]	KSL-1	3.4	-20.6	1.3	Ungulates
Kesslerloch	*Equus ferus*		Bocherens et al.[79]	KSL-2	3.4	-20.4	1.6	Ungulates
Kesslerloch	*Equus ferus*		Bocherens et al.[79]	KSL-3	3.4	-20.2	2.3	Ungulates
Kesslerloch	*Equus ferus*		Bocherens et al.[79]	KSL-4	3.4	-20.3	0.6	Ungulates
Kesslerloch	*Equus ferus*		Bocherens et al.[79]	KSL-5	3.4	-20.0	1.7	Ungulates
Geißenklösterle	*Lepus sp.*		Baumann et al.[80]	VLP-15	3.3	-20.8	1.9	Small game
Kesslerloch	*Lepus timidus*		Bocherens et al.[79]	KSL-19	3.1	-20.8	0.9	Small game
Kesslerloch	*Lepus timidus*		Bocherens et al.[79]	KSL-20	3.2	-20.2	0.7	Small game
Kesslerloch	*Lepus timidus*		Bocherens et al.[79]	KSL-22	3.3	-20.8	0.5	Small game
Kesslerloch	*Lepus timidus*		Bocherens et al.[79]	KSL-23	3.0	-21.4	0.2	Small game
Kesslerloch	*Lepus timidus*		Bocherens et al.[79]	KSL-24	3.0	-20.6	0.3	Small game
Kesslerloch	*Lepus timidus*		Bocherens et al.[79]	KSL-25	3.3	-20.4	2.3	Small game
Kesslerloch	*Lepus timidus*		Bocherens et al.[79]	KSL-26	3.0	-19.7	1.3	Small game
Kesslerloch	*Lepus timidus*		Bocherens et al.[79]	KSL-27	3.4	-20.2	0.4	Small game
Kesslerloch	*Lepus timidus*		Bocherens et al.[79]	KSL-28	3.3	-20.4	0.6	Small game
Kesslerloch	*Lepus timidus*		Bocherens et al.[79]	KSL-29	3.1	-20.5	1.1	Small game
Kesslerloch	*Lepus timidus*		Bocherens et al.[79]	KSL-30	3.1	-20.2	0.5	Small game

Kesslerloch	*Lepus timidus*		Bocherens et al.[79]	KSL-31	3.0	-20.1	1.9	Small game
Kesslerloch	*Lepus timidus*		Bocherens et al.[79]	KSL-32	3.1	-20.4	1.0	Small game
Kesslerloch	*Lepus timidus*		Bocherens et al.[79]	KSL-33	3.0	-20.3	1.6	Small game
Kesslerloch	*Lepus timidus*		Bocherens et al.[79]	KSL-34	2.9	-20.2	0.9	Small game
Kesslerloch	*Lepus timidus*		Bocherens et al.[79]	KSL-35	3.3	-20.9	1.5	Small game
Kesslerloch	*Mammuthus primigenius*		Bocherens et al.[79]	KSL-6	3.3	-21.3	5.0	Megafauna
Kesslerloch	*Mammuthus primigenius*		Bocherens et al.[79]	KSL-47	3.2	-20.5	6.4	Megafauna
Hohle Fels	*Megaloceros giganteus*		Immel et al.[112]	HF-15	3.3	-20.1	2.9	Megafauna
Geißenklösterle	*Rangifer tarandus*		Stevens & Hedges[114]	OxA-6254	3.0	-19.4	0.1	Ungulates
Geißenklösterle	*Rangifer tarandus*		Bocherens et al.[79]	GSK-1	3.2	-19.6	2.0	Ungulates
Hohle Fels	*Rangifer tarandus*		Bocherens et al.[79]	HFL-1	3.2	-19.4	1.0	Ungulates
Kesslerloch	*Rangifer tarandus*		Drucker et al.[113]	RA-KSL-620	3.3	-19.1	2.4	Ungulates
Kesslerloch	*Rangifer tarandus*		Drucker et al.[113]	RA-KSL-628	3.4	-19.9	2.6	Ungulates
Kesslerloch	*Rangifer tarandus*		Drucker et al.[113]	RA-KSL-632	3.4	-19.8	2.7	Ungulates
Kesslerloch	*Rangifer tarandus*		Drucker et al.[113]	RA-KSL-633	3.3	-20.3	2.9	Ungulates
Kesslerloch	*Rangifer tarandus*		Drucker et al.[113]	RA-KSL-635	3.3	-19.9	2.8	Ungulates
Kesslerloch	*Spermophilus major*		Bocherens et al.[79]	KSL-37	3.5	-20.9	1.7	Small game
Kesslerloch	*Spermophilus major*		Bocherens et al.[79]	KSL-38	3.3	-20.3	2.0	Small game
Kesslerloch	*Spermophilus major*		Bocherens et al.[79]	KSL-39	3.3	-21.0	2.5	Small game
Kesslerloch	*Spermophilus major*		Bocherens et al.[79]	KSL-40	3.4	-20.8	2.2	Small game
Risch-Rotkreuz	*Mammuthus primigenius*		Drucker et al.[115]	ZUG-1	3.3	-21.1	6.2	Megafauna
Petersfels	*Rangifer tarandus*		Drucker et al.[113]	RA-PTF 363	3.4	-19.8	2.4	Ungulates
Petersfels	*Rangifer tarandus*		Drucker et al.[113]	RA-PTF 364	3.4	-19.6	2.1	Ungulates
Petersfels	*Rangifer tarandus*		Drucker et al.[113]	RA-PTF 365	3.4	-19.9	2.3	Ungulates
Petersfels	*Rangifer tarandus*		Drucker et al.[113]	RA-PTF 366	3.2	-20.0	1.2	Ungulates
Petersfels	*Rangifer tarandus*		Drucker et al.[113]	RA-PTF 367	3.2	-19.9	1.2	Ungulates
Petersfels	*Rangifer tarandus*		Drucker et al.[113]	RA-PTF 368	3.3	-19.4	2.4	Ungulates
Petersfels	*Rangifer tarandus*		Drucker et al.[113]	RA-PTF 369	3.3	-20.2	2.6	Ungulates
Petersfels	*Rangifer tarandus*		Drucker et al.[113]	RA-PTF 370	3.4	-19.1	2.3	Ungulates
Petersfels	*Rangifer tarandus*		Drucker et al.[113]	RA-PTF 371	3.5	-19.7	2.1	Ungulates

Petersfels	*Rangifer tarandus*		Drucker et al.[113]	RA-PTF 373	3.4	-19.7	2.5	Ungulates
Petersfels	*Rangifer tarandus*		Drucker et al.[113]	RA-PTF 374	3.4	-19.7	1.4	Ungulates
Petersfels	*Rangifer tarandus*		Drucker et al.[113]	RA-PTF 375	3.4	-20.0	3.3	Ungulates
Petersfels	*Rangifer tarandus*		Drucker et al.[113]	RA-PTF 376	3.4	-19.7	2.4	Ungulates
Petersfels	*Rangifer tarandus*		Drucker et al.[113]	RA-PTF 377	3.4	-19.9	2.0	Ungulates
Petersfels	*Rangifer tarandus*		Drucker et al.[113]	RA-PTF 379	3.5	-19.5	2.6	Ungulates
Petersfels	*Rangifer tarandus*		Drucker et al.[113]	RA-PTF 380	3.6	-19.6	2.4	Ungulates
Petersfels	*Rangifer tarandus*		Drucker et al.[113]	RA-PTF 381	3.6	-19.3	2.3	Ungulates
Petersfels	*Rangifer tarandus*		Drucker et al.[113]	RA-PTF 382	3.4	-19.1	1.9	Ungulates
Petersfels	*Rangifer tarandus*		Drucker et al.[113]	RA-PTF 383	3.6	-19.4	2.9	Ungulates
Schussenquelle	*Rangifer tarandus*		Drucker et al.[113]	SCH-1	3.4	-19.8	2.5	Ungulates
Schussenquelle	*Rangifer tarandus*		Drucker et al.[113]	SCH-2	3.3	-19.7	2.8	Ungulates
Schussenquelle	*Rangifer tarandus*		Drucker et al.[113]	SCH-3	3.4	-20.1	1.8	Ungulates
Schussenquelle	*Rangifer tarandus*		Drucker et al.[113]	SCH-4	3.3	-19.4	1.9	Ungulates
Schussenquelle	*Rangifer tarandus*		Drucker et al.[113]	SCH-5	3.5	-19.5	2.2	Ungulates

Table S6: Results of the molecular dating of the samples that are not radiocarbon dated. Estimation of the median age, as well as the values of the 95% HPD interval estimates low and high are given. The age is given in years cal BP.

Short-ID	median	95% HPD interval Low Estimate	95% HPD interval High Estimate
F-1986.2	5190	220	10740
F-1986.1	7908	487	15582
F-α19496	7926	6	17347
UA-206	10620	2254	19400
UA-208	9084	1340	17685
UA-207	9080	1445	17819
UA-203	7078	10	14166
HF-912*	42213	26863	57688
HF-1174	32728	15981	48807
HF-1553	49036	34994	65197
HF-1712	40325	21909	57416

*The sample was 14C dated to 31586 – 31136 cal yBP at later stages of the manuscript preparation. However, due to the proximity of the 14C age to the phylogenetically estimated age, we did not redo the BEAST estimation including the additional date.

Table S7: Estimation of nucleotide diversity and standard deviations (SD) of the defined population groups. Gnirshöhle canids are from Southern Germany. "GN" defines the population from Gnirshöhle. The population “KSL" represent the Magdalenian canids from Kesslerloch. From previous studies[4,93,105,106] mitochondrial canid genomes are grouped in "mWolves" and "mDogs". Genetic diversity, "Nucleotide Diversity (Nuc_Div)," was estimated regarding the complete mitochondrial genome by DnaSP[116] v5 by excluding all presented gaps for each population group individually.

Population	Population ID	Population size n	total number of sites	Mean Nuc_Div Pi	SD Nuc_Div
Gnirshöhle Canids	GN	5	11710	0.00306	0.00106
Kesslerloch Canids	KSL	5	13390	0.00379	0.00098
Modern Wolves	mWolves	75	15316	0.00501	0.00101
Modern Dogs	mDogs	79	15804	0.00339	0.00021

Table S8: Information about the assignment of the canid mitochondrial DNA sequences into defined canid population groups.

Population Group Name	canid mitochondrial DNA sequences
Gnirshöhle Canids	GN-133_15ka, GN-192_Mag, GN-14_15.5ka, GN-106_15.5ka, GN-999_15ka
Kesslerloch Canids	KSL-60_14.5ka, KSL-62_14.5ka, KSL-58_14.5ka, KSL-189_14ka, KSL-61_14ka
Modern Wolves	China1, Mongolia1, China2, Sweden1, Saudi_Arabia1, Saudi_Arabia2, Spain1, Japan1, Finland, Russia1, Sweden2, China3, Israel1, India1, Russia2, Poland1, Russia3, Ukraine, Italy, Poland2, Oman, Iran, Sweden3, China4, Croatia, Israe2, Canada1, Canada2, Alaska1, Canada3, Canada4, Mexico1, Canada5, Canada6, Canada7, USA1, Mexico2, Alaska2, Alaska3, USA2, USA3, USA4, Alaska4, Alaska5, Alaska6, Canada8, Canada9, Canada10, Canada11, China5, China6, Japan11, Mongolia2, Canada12, Canada13, Canada14, Canada15, Canada16, Canada17, Canada18, Canada19, Canada20, Canada21, Afghanistan, Syria, Mexico3, Spain2, India2, Denmark, Mongolia3, Saudi_Arabia3, Turkey, Alaska7, Alaska8, Alaska9
modern Dog	ChineseDog1, ChineseDog2, ChineseDog3, Dingo, D01, D03, D04, D05, D07, D09, D11, D12, D15, D16, D18, D21, D22, D25, D26, D27, D28, D30, D31, D32, D33, D34, D35, D40, D41, D42, D44, D46, D47, D48, D49, D50, D51, D52, D55, D56, D59, D60, D61, D63, D65, D66, D67, D68, D69, D71, D72, D73, D75, D78, D79, D80, D81, D82, D83, D84, D85, D86, D87, D88, D89, D90, D91, D93, D94, D95, D96, D97, D98, D99, D102, D103, Basenji, Japan4, Japan10

Table S9: Information about the sequences included in the multiple sequence alignment used for genetic analyses performed in this study. The table provides information about the sample ID used in the phylogenetic tree, the mitochondrial genome coverage, the reference of the published genomes (see numbers 1 to 20 in table[4,93,98,104-106,116-125]) and the corresponding accession numbers, if the samples are either ancient or modern. Further the table provides information about the dating of the samples, including the reference for the dating (see numbers 1 to 4 and 20 to 37 in table[4,53,55,61,93,105,106,126-138]) as well as the information about the examined canid.

(Separate file)

References

1 Rütimeyer, L. *Die Knochenhöhle von Thayingen bei Schaffhausen*. (F. Vieweg & Sohn, 1875).

2 Ivy-Ochs, S., Schäfer, J., Kubik, P. W., Synal, H.-A. & Schlüchter, C. Timing of deglaciation on the northern Alpine foreland (Switzerland). *Eclogae Geologicae Helvetiae* **97**, 47-55 (2004).

3 Napierala, H. *Die Tierknochen aus dem Kesslerloch: Neubearbeitung der paläolithischen Fauna*. (Baudepartement des Kantons Schaffhausen, Kantonsarchäologie Schaffhausen, 2008).

4 Thalmann, O. *et al.* Complete Mitochondrial Genomes of Ancient Canids Suggest a European Origin of Domestic Dogs. *Science* **342**, 871-874 (2013).

5 Peters, E., Praeger, W., Vogel, R. & von Mandach, E. *Die altsteinzeitliche Kulturstätte Petersfels*. (Dr. B. Filser, 1930).

6 Peters, E. & Toepfer, V. *Der Abschluss der Grabungen am Petersfels bei Engen im badischen Hegau*. Vol. 23 155-198 (Prähistorische Zeitschrift 1932).

7 Albrecht, G. *Magdalénien-Inventare vom Petersfels: siedlungsarchäologische Ergebnisse der Ausgrabungen 1974-1976*. Vol. 6 (Verlag Archaeologica Venatoria, 1979).

8 Albrecht, G. in *Urgeschichte in Baden-Württemberg* (ed Hansjürgen Müller-Beck) 331-353 (Theiss Verlag, 1983).

9 Albrecht, G., Drautz, D. & Kind, J. Eine Station des Magdalénien in der Gnirshöhle bei Engen- Bittelbrunn im Hegau. *Archäologisches Korrespondenzblatt* **7**, 161-179 (1977).

10 Albrecht, G., Wong, G. L. & Münzel, S. C. in *„All der holden Hügel ist keiner mir fremd..." Festschrift zum 65. Geburtstag von Claus-Joachim Kind.* (eds M. Baales & C. Pasda) 301-310 (Archaeologica Venatoria, 2019).

11 Albrecht, G. & Berke, H. in *De la Loire à l'Oder BAR Int. Series* (ed Marcel Otte) 465-473 (1988).

12 Münzel, S. C. The faunal assemblage of Gnirshöhle. (in prep.).

13 Stuart, A. J. & Lister, A. M. New radiocarbon evidence on the extirpation of the spotted hyaena (Crocuta crocuta (Erxl.)) in northern Eurasia. *Quaternary Science Reviews* **96**, 108-116 (2014).

14 Haynes, G. Utilization and skeletal disturbances of North American prey carcasses. *Arctic*, 266-281 (1982).

15 Conard, N. J., Kind, C.-J., Kretschmann, W., Wolf, C. & Engler, B. *Als der Mensch die Kunst erfand*. (Theiss, 2017).

16 Conard, N. J. & Bolus, M. Radiocarbon dating the appearance of modern humans and timing of cultural innovations in Europe: new results and new challenges. *Journal of human Evolution* **44**, 331-371 (2003).

17 Conard, N. J. & Bolus, M. Radiocarbon dating the late Middle Paleolithic and the Aurignacian of the Swabian Jura. *Journal of Human Evolution* **55**, 886-897 (2008).

18 Higham, T. *et al.* Testing models for the beginnings of the Aurignacian and the advent of figurative art and music: The radiocarbon chronology of Geißenklösterle. *Journal of human evolution* **62**, 664-676 (2012).

19 Conard, N. J., Bolus, M., Dutkiewicz, E. & Wolf, S. Eiszeitarchäologie auf der Schwäbischen Alb. *Die Fundstellen im Ach-und Lonetal und in ihrer Umgebung. Tübingen: Kerns Verlag* (2015).

20 Hahn, J. Neue Grabungen im Hohlen Felsen bei Schelklingen, Alb-Donau-Kreis. *Archäologische Ausgrabungen in Baden-Württemberg 1988*, 20-22 (1989).

21 Langguth, K. & Malina, M. in *Eiszeitschmuck - Status und Schönheit* Vol. Museumsheft 6 (eds S. Köbl & N. J. Conard) 93-128 (Urgeschichtliches Museum, 2003).

22 Conard, N. J. A female figurine from the basal Aurignacian of Hohle Fels Cave in southwestern Germany. *Nature* **459**, 248-252 (2009).

23 Wolf, S. *Schmuckstücke: Die Elfenbeinbearbeitung im Schwäbischen Aurignacien*. (Kerns Verlag, 2015).
24 Wolf, S. & Conard, N. J. Personal Ornaments of the Swabian Aurignacian. *Palethnologie. Archéologie et sciences humaines* (2015).
25 Conard, N. J., Malina, M. & Münzel, S. C. New flutes document the earliest musical tradition in southwestern Germany. *Nature* **460**, 737-740 (2009).
26 Münzel, S. C., Conard, N. J., Hein, W., Gill, F. & Potengowski, A. F. in *Studien zur Musikarchäologie X Orient-Archäologie* (eds R Eichmann, F Jianjun, & L.-Ch Koch) 225-243 (Verlag Marie Leidorf GmbH, 2016).
27 Münzel, S. C. & Conard, N. J. Cave bear hunting in the Hohle Fels, a cave site in the Ach Valley, Swabian Jura. *Revue de Paléobiologie* **23**, 877-885 (2004).
28 Conard, N. J., Kitagawa, K., Krönneck, P., Böhme, M. & Münzel, S. C. in *Zooarchaeology and Modern Human Origins Vertebrate Paleobiology and Paleoanthropology* Ch. Chapter 11, 173-190 (2013).
29 Napierala, H., Münzel, S. C. & Conard, N. J. in *Das Magdalénien des Hohle Fels. Chronologische Stellung, Lithische Technologie und Funktion der Rückenmesser* (ed Andreas Taller) 275-311 (Kerns Verlag, 2014).
30 Stuart, A. J. & Lister, A. M. Extinction chronology of the cave lion Panthera spelaea. *Quaternary Science Reviews* **30**, 2329-2340 (2011).
31 Çep, B. & Krönneck, P. Landscape and cave use in the Middle Paleolithic of Bockstein: new results from the lithic and fauna analysis. *Settlement Dynamics of the Middle Palaeolithic and Middle Stone Age IV*, 227-251 (2015).
32 Krönneck, P. *Die pleistozäne Makrofauna des Bocksteins (Lonetal–Schwäbische Alb). Ein neuer Ansatz zur Rekonstruktion der Paläoumwelt*, Ph. D. Dissertation, University of Tübingen, (2012).
33 Krönneck, P., Niven, L. & Uerpmann, H. P. Middle Palaeolithic subsistence in the Lone Valley (Swabian Alb, southern Germany). *International Journal of Osteoarchaeology* **14**, 212-224 (2004).
34 Taylor, W. E. Summary of archaeological field work on Banks and Victoria Islands, Arctic Canada, 1965. *Arctic Anthropology* **4**, 221-243 (1967).
35 Müller-Beck, H. *Excavations at Umingmak on Banks Island, NWT, 1970 and 1973 preliminary report*. (1977).
36 Owen, L. *The Microblades of Umingmak. Archäologische Forschungen auf Banks Island 1970-1975*. (Archaeologica Venatoria, 1984).
37 Münzel, S. C. *Umingmak, ein Moschusochsenjagdplatz auf Banks Island, N.W.T., Canada. Archäozoologische Auswertung des Areals ID*. Vol. 5,2 (Archaeologica Venatoria, 1987).
38 Albrecht, G. in *Spuren der Jagd - Die Jagd nach Spuren. Festschrift für H. Müller-Beck Tübinger Monographien zur Urgeschichte* (eds Ingo Campen, J Hahn, & Hans-Peter Uerpmann) (1996).
39 Müller-Beck, H. in *Kanada in der geographischen Forschung der 80er Jahre Kanada-Studien* (ed R. Vogelsang) 19-35 (1989).
40 Münzel, S. C. Quantitative analysis and archaeological site interpretation. *Archaeozoologia* **2**, 93-109 (1988).
41 Münzel, S. Seasonal activities at Umingmak a Muskox-Hunting site on banks island, NWT, Canada, with special reference to the bird remains. *Animals and Archaeology* **1**, 249-257 (1983).
42 Haidle, M. *Der menschliche Unterkiefer von Umingmak. Morphologie und Funktion*, unpublished MA thesis, Institut für Urgeschichte, University of Tübingen, (1991).
43 Bocherens, H. *et al.* Isotopic evidence (C, N, S) for a high aquatic dietary contribution for a Pre-Dorset muskox hunter from Umingmak (Banks Island, Canada). *Journal of Archaeological Science: Reports* **6**, 700-708, doi:10.1016/j.jasrep.2015.08.021 (2016).

44 Verjux, C. *Les structures en creux du site mésolithique d'Auneau" le Parc du Château"(Eure-et-Loir): nouveau bilan et implications concernant le mode de vie des dernières populations de chasseurs-collecteurs en Europe*, Paris 1, (2015).
45 Verjux, C. in *Creuser au Mésolithique, Actes de la séance de la Société préhistorique française (Châlons-en-Champagne, mars 2016) Séances de la Société préhistorique française* (eds N Achard-Corompt, E Ghesquière, & V Riquier) 155-172 (Société préhistorique française, 2017).
46 Leduc, C. & Verjux, C. Mesolithic occupation patterns at Auneau "Le Parc du Château"(Eure-et-Loir–France): contribution of zooarchaeological analysis from two main pits to the understanding of type and length of occupation. *Journal of archaeological science* **47**, 39-52 (2014).
47 Leduc, C. & Verjux, C. in *Au cœur des sites mésolithiques : entre processus taphonomiques et données archéologiques, Actes de la table-ronde internationale de Besançon (Doubs, France), « Hommages au Professeur André Thévenin » 29-30 octobre 2013 Annales Littéraires 983 « Environnement, sociétés et archéologie »* (eds Cupillard C, Griselin S, & Séara F) 79-96 (Besançon, Presses universitaires de Franche-Comté, 2018).
48 Fischer, U. Grabungen im romischen Steinkastell von Heddernheim 1957-1959 (mit Beitragen von Kurt DEPPERT, Charlotte FISCHER und Ingeborg HULD-ZETSCHE). *Schriften des Frankfurter Museums für Vor-und Frühgeschichte II*, 54–55. 252 (1973).
49 Hahn, E. in *Das Fränkische Gräberfeld von Nieder-Erlenbach, Stadt Frankfurt am Main Beiträge zum Denkmalschutz in Frankfurt am Main* (ed M Dohrn-Ihmig) 297-320 (1999).
50 Dohrn-Ihmig, M. & Döry, L. in *Frankfurter Fundchronik der Jahre 1980–1986 Schriften des Frankfurter Museums* 162-168 (1987).
51 Ament, H. Zur archäologischen Periodisierung der Merowingerzeit. *Germania* **55**, 133-140 (1977).
52 Böhner, K. *Die frankischen Altertümer des Trierer Ländes*. (1958).
53 Germonpré, M. *et al.* Fossil dogs and wolves from Palaeolithic sites in Belgium, the Ukraine and Russia: osteometry, ancient DNA and stable isotopes. *Journal of Archaeological Science* **36**, 473-490, doi:10.1016/j.jas.2008.09.033 (2009).
54 Germonpré, M., Lázničková-Galetová, M. & Sablin, M. V. Palaeolithic dog skulls at the Gravettian Předmostí site, the Czech Republic. *Journal of Archaeological Science* **39**, 184-202, doi:https://doi.org/10.1016/j.jas.2011.09.022 (2012).
55 Germonpré, M., Lázničková-Galetová, M., Losey, R. J., Räikkönen, J. & Sablin, M. V. Large canids at the Gravettian Předmostí site, the Czech Republic: The mandible. *Quaternary International* **359-360**, 261-279, doi:https://doi.org/10.1016/j.quaint.2014.07.012 (2015).
56 Perri, A. A wolf in dog's clothing: Initial dog domestication and Pleistocene wolf variation. *Journal of Archaeological Science* **68**, 1-4, doi:10.1016/j.jas.2016.02.003 (2016).
57 Janssens, L. A. A. *From wolf to dog*, uitgever niet vastgesteld, (2019).
58 Ameen, C. *et al.* A landmark-based approach for assessing the reliability of mandibular tooth crowding as a marker of dog domestication. *Journal of Archaeological Science* **85**, 41-50, doi:10.1016/j.jas.2017.06.014 (2017).
59 Nobis, G. Die Wildsäugetiere in der Umwelt des Menschen von Oberkassel bei Bonn und das Domestikationsproblem von Wölfen im Jungpaläolithikum. *Bonner Jahrbücher*, 367-376 (1986).
60 Janssens, L. *et al.* A new look at an old dog: Bonn-Oberkassel reconsidered. *Journal of Archaeological Science* **92**, 126-138, doi:https://doi.org/10.1016/j.jas.2018.01.004 (2018).
61 Napierala, H. & Uerpmann, H.-P. A 'new' palaeolithic dog from central Europe. *International Journal of Osteoarchaeology* **22**, 127-137, doi:10.1002/oa.1182 (2012).

62 Münzel, S. C. in *Geißenklösterle: Chronostratigraphie, Paläoumwelt und Subsistenz im Mittel- und Jungpaläolithikum der Schwäbischen Alb* (eds Nicholas J. Conard, M. Bolus, & Susanne C. Münzel) 147-327 (Kerns Verlag, 2019).
63 Camarós, E., Münzel, S. C., Cueto, M., Rivals, F. & Conard, N. J. The evolution of Paleolithic hominin–carnivore interaction written in teeth: Stories from the Swabian Jura (Germany). *Journal of Archaeological Science: Reports* **6**, 798-809, doi:10.1016/j.jasrep.2015.11.010 (2016).
64 Prassack, K. A., DuBois, J., Lázničková-Galetová, M., Germonpré, M. & Ungar, P. S. Dental microwear as a behavioral proxy for distinguishing between canids at the Upper Paleolithic (Gravettian) site of Předmostí, Czech Republic. *Journal of Archaeological Science* **115**, 105092 (2020).
65 Hajdas, I., Bonani, G., Furrer, H., Mäder, A. & Schoch, W. Radiocarbon chronology of the mammoth site at Niederweningen, Switzerland: results from dating bones, teeth, wood, and peat. *Quaternary International* **164**, 98-105 (2007).
66 Hajdas, I. Radiocarbon dating and its applications in Quaternary studies. *Eiszeitalter und Gegenwart Quaternary Science Journal* **57**, 24 (2008).
67 Reimer, P. J. *et al.* IntCal13 and Marine13 radiocarbon age calibration curves 0–50,000 years cal BP. *Radiocarbon* **55**, 1869-1887 (2013).
68 Bronk Ramsey, C. (Oxford Radiocarbon Accelerator Unit: University of Oxford. Available at …, 2017).
69 Bronk Ramsey, C. Methods for summarizing radiocarbon datasets. *Radiocarbon* **59**, 1809-1833 (2017).
70 Kind, C.-J. Die absolute Datierung des Magdaléniens und des Mesolithikums in Süddeutschland. *Erkenntnisjäger. Kultur und Umwelt des Frühen Menschen. Festschrift D. Mania. Veröffentlichungen des Landesamtes für Archäologie Sachsen-Anhalt. Landesmuseum für Vorgeschichte* **57**, 303-219 (2003).
71 Taller, A. *Das Magdalénien des Hohle Fels. Chronologische Stellung, Lithische Technologie und Funktion der Rückenmesser*. (Kerns Verlag, 2014).
72 Hornauer-Jahnke, T. K. & Noack, E. S. in *„All der holden Hügel ist keiner mir fremd…" Festschrift zum 65. Geburtstag von Claus-Joachim Kind.* (eds M. Baales & C. Pasda) 293-314 (Archaeologica Venatoria, 2019).
73 Bocherens, H., Drucker, D., Billiou, D. & Moussa, I. Une nouvelle approche pour évaluer l'état de conservation de l'os et du collagène pour les mesures isotopiques (datation au radiocarbone, isotopes stables du carbone et de l'azote). *l'Anthropologie* **109**, 557-567 (2005).
74 Bocherens, H. *et al.* Paleobiological Implications of the Isotopic Signatures (13C, 15N) of Fossil Mammal Collagen in Scladina Cave (Sclayn, Belgium). *Quaternary Research* **48**, 370-380 (1997).
75 DeNiro, M. J. Postmortem preservation and alteration of in vivo bone collagen isotope ratios in relation to palaeodietary reconstruction. *Nature* **317**, 806, doi:10.1038/317806a0 (1985).
76 Ambrose, S. H. Preparation and characterization of bone and tooth collagen for isotopic analysis. *Journal of Archaeological Science* **17**, 431-451, doi:https://doi.org/10.1016/0305-4403(90)90007-R (1990).
77 Jackson, A. L., Inger, R., Parnell, A. C. & Bearhop, S. Comparing isotopic niche widths among and within communities: SIBER - Stable Isotope Bayesian Ellipses in R. *Journal of Animal Ecology* **80**, 595-602, doi:10.1111/j.1365-2656.2011.01806.x (2011).
78 Layman, C. A., Arrington, D. A., Montaña, C. G. & Post, D. M. Can isotope ratios provide for community-wide measures of trophic structure? *Ecology* **88**, 42-48 (2007).
79 Bocherens, H. *et al.* Isotopic evidence for dietary ecology of cave lion (Panthera spelaea) in North-Western Europe: Prey choice, competition and implications for extinction. *Quaternary International* **245**, 249-261, doi:10.1016/j.quaint.2011.02.023 (2011).

80 Baumann, C. *et al.* Dietary niche partitioning among Magdalenian canids in southwestern Germany and Switzerland *Quaternary Science Reviews* **227**, 106032 (2020).
81 Stock, B. C. & Semmens, B. X. MixSIAR GUI User Manual v3.1. (2016).
82 Bocherens, H. Isotopic tracking of large carnivore palaeoecology in the mammoth steppe. *Quaternary Science Reviews* **117**, 42-71, doi:10.1016/j.quascirev.2015.03.018 (2015).
83 Krajcarz, M. T., Krajcarz, M. & Bocherens, H. Collagen-to-collagen prey-predator isotopic enrichment (Δ 13 C, Δ 15 N) in terrestrial mammals - a case study of a subfossil red fox den. *Palaeogeography, Palaeoclimatology, Palaeoecology* **490**, 563-570, doi:10.1016/j.palaeo.2017.11.044 (2018).
84 Dionne, K., Dufresne, F. & Nozais, C. Variation in δ13C and δ15N trophic enrichment factors among Hyalella azteca amphipods from different lakes. *Hydrobiologia* **781**, 217-230 (2016).
85 Gelman, A. *et al. Bayesian data analysis*. (Taylor & Francis, 2014).
86 Cooper, A. & Poinar, H. N. Ancient DNA: do it right or not at all. *Science* **289**, 1139-1139 (2000).
87 Knapp, M. & Hofreiter, M. Next generation sequencing of ancient DNA: requirements, strategies and perspectives. *Genes* **1**, 227-243 (2010).
88 Dabney, J. *et al.* Complete mitochondrial genome sequence of a Middle Pleistocene cave bear reconstructed from ultrashort DNA fragments. *Proceedings of the National Academy of Sciences* **110**, 15758-15763 (2013).
89 Meyer, M. & Kircher, M. Illumina sequencing library preparation for highly multiplexed target capture and sequencing. *Cold Spring Harbor Protocols* **2010**, pdb. prot5448 (2010).
90 Kircher, M. in *Ancient DNA* 197-228 (Springer, 2012).
91 Maricic, T., Whitten, M. & Pääbo, S. Multiplexed DNA sequence capture of mitochondrial genomes using PCR products. *PloS one* **5**, e14004 (2010).
92 Furtwängler, A. *et al.* Ratio of mitochondrial to nuclear DNA affects contamination estimates in ancient DNA analysis. *Scientific reports* **8**, 1-8 (2018).
93 Loog, L. *et al.* Ancient DNA suggests modern wolves trace their origin to a late Pleistocene expansion from Beringia. *Molecular ecology*, 1-15 (2019).
94 Peltzer, A. *et al.* EAGER: efficient ancient genome reconstruction. *Genome biology* **17**, 60 (2016).
95 Andrews, S. (Babraham Bioinformatics, Babraham Institute, Cambridge, United Kingdom, 2010).
96 Schubert, M., Lindgreen, S. & Orlando, L. AdapterRemoval v2: rapid adapter trimming, identification, and read merging. *BMC research notes* **9**, 1-7 (2016).
97 *http://broadinstitute.github.io/picard/*.
98 Kim, K. S., Lee, S. E., Jeong, H. W. & Ha, J. H. The complete nucleotide sequence of the domestic dog (Canis familiaris) mitochondrial genome. *Molecular phylogenetics and evolution* **10**, 210-220 (1998).
99 Ginolhac, A., Rasmussen, M., Gilbert, M. T. P., Willerslev, E. & Orlando, L. mapDamage: testing for damage patterns in ancient DNA sequences. *Bioinformatics* **27**, 2153-2155 (2011).
100 Okonechnikov, K., Conesa, A. & García-Alcalde, F. Qualimap 2: advanced multi-sample quality control for high-throughput sequencing data. *Bioinformatics* **32**, 292-294 (2016).
101 McKenna, A. *et al.* The Genome Analysis Toolkit: a MapReduce framework for analyzing next-generation DNA sequencing data. *Genome research* **20**, 1297-1303 (2010).
102 Skoglund, P. *et al.* Separating endogenous ancient DNA from modern day contamination in a Siberian Neandertal. *Proceedings of the National Academy of Sciences* **111**, 2229-2234 (2014).
103 Nguyen, L.-T., Schmidt, H. A., Von Haeseler, A. & Minh, B. Q. IQ-TREE: a fast and effective stochastic algorithm for estimating maximum-likelihood phylogenies. *Molecular biology and evolution* **32**, 268-274 (2015).

104 Skoglund, P., Ersmark, E., Palkopoulou, E. & Dalén, L. Ancient wolf genome reveals an early divergence of domestic dog ancestors and admixture into high-latitude breeds. *Current Biology* **25**, 1515-1519 (2015).
105 Frantz, L. A. *et al.* Genomic and archaeological evidence suggest a dual origin of domestic dogs. *Science* **352**, 1228-1231 (2016).
106 Botigué, L. R. *et al.* Ancient European dog genomes reveal continuity since the Early Neolithic. *Nature communications* **8**, 1-11 (2017).
107 Suchard, M. A. *et al.* Bayesian phylogenetic and phylodynamic data integration using BEAST 1.10. *Virus evolution* **4**, vey016 (2018).
108 Baumann, C., Bocherens, H., Drucker, D. G. & Conard, N. J. Fox dietary ecology as a tracer of human impact on Pleistocene ecosystems. *PLOS ONE* **15**, e0235692, doi:10.1371/journal.pone.0235692 (2020).
109 Von den Driesch, A. *A guide to the measurement of animal bones from archaeological sites: as developed by the Institut für Palaeoanatomie, Domestikationsforschung und Geschichte der Tiermedizin of the University of Munich*. Vol. 1 (Peabody Museum Press, 1976).
110 Boessneck, J., von den Driesch, A., Lepiksaar, J., Riek, G. & Storch, G. *Das Paläolithikum der Brillenhöhle bei Blaubeuren (Schwäbische Alb) II: Die jungpleistozänen Tierknochenfunde aus der Brillenhöhle*. (Verlag Müller & Gräff, 1973).
111 Drucker, D. G., Bridault, A., Cupillard, C., Hujic, A. & Bocherens, H. Evolution of habitat and environment of red deer (Cervus elaphus) during the Late-glacial and early Holocene in eastern France (French Jura and the western Alps) using multi-isotope analysis (δ13C, δ15N, δ18O, δ34S) of archaeological remains. *Quaternary International* **245**, 268-278, doi:10.1016/j.quaint.2011.07.019 (2011).
112 Immel, A. *et al.* Mitochondrial Genomes of Giant Deers Suggest their Late Survival in Central Europe. *Sci Rep* **5**, 10853, doi:10.1038/srep10853 (2015).
113 Drucker, D. G., Kind, C. J. & Stephan, E. Chronological and ecological information on Late-glacial and early Holocene reindeer from northwest Europe using radiocarbon (14C) and stable isotope (13C, 15N) analysis of bone collagen: Case study in southwestern Germany. *Quaternary International* **245**, 218-224, doi:10.1016/j.quaint.2011.05.007 (2011).
114 Stevens, R. E. & Hedges, R. E. M. Carbon and nitrogen stable isotope analysis of northwest European horse bone and tooth collagen, 40,000BP–present: Palaeoclimatic interpretations. *Quaternary Science Reviews* **23**, 977-991, doi:10.1016/j.quascirev.2003.06.024 (2004).
115 Drucker, D. G., Furtwängler, A., Schünemann, V., Huber, R. & Reinhard, J. Durchleuchtet und analysiert. Ein Update zur Genetik, Isotopie und Radiografie des «letzten Zuger Mammuts». *TUGIUM* **34**, 1-9 (2018).
116 Wang, G.-d. *et al.* The genomics of selection in dogs and the parallel evolution between dogs and humans. *Nature communications* **4**, 1-9 (2013).
117 Björnerfeldt, S., Webster, M. T. & Vilà, C. Relaxation of selective constraint on dog mitochondrial DNA following domestication. *Genome research* **16**, 990-994 (2006).
118 Shahid, S. A. *A survey of the canine and bovine mitochondrial genomes*. (University of Missouri-Columbia, 2004).
119 Webb, K. M. & Allard, M. W. Mitochondrial genome DNA analysis of the domestic dog: identifying informative SNPs outside of the control region. *Journal of forensic sciences* **54**, 275-288 (2009).
120 Baranowska, I. *et al.* Sensory ataxic neuropathy in Golden Retriever dogs is caused by a deletion in the mitochondrial tRNA Tyr gene. *PLoS Genet* **5**, e1000499 (2009).
121 Meng, C., Zhang, H. & Meng, Q. Mitochondrial genome of the Tibetan wolf. *Mitochondrial DNA* **20**, 61-63 (2009).

122 Arnason, U., Gullberg, A., Janke, A. & Kullberg, M. Mitogenomic analyses of caniform relationships. *Molecular phylogenetics and evolution* **45**, 863-874 (2007).

123 Matsumura, S., Inoshima, Y. & Ishiguro, N. Reconstructing the colonization history of lost wolf lineages by the analysis of the mitochondrial genome. *Molecular phylogenetics and evolution* **80**, 105-112 (2014).

124 Zhang, H., Zhang, J., Chen, L. & Liu, G. (Taylor & Francis, 2014).

125 Zhang, H. *et al.* Complete mitochondrial genome of Canis lupus campestris. *Mitochondrial DNA* **26**, 255-256 (2015).

126 Leonard, J. A. *et al.* Megafaunal extinctions and the disappearance of a specialized wolf ecomorph. *Current Biology* **17**, 1146-1150 (2007).

127 Kandel, A., Gasparyan, B., Bruch, A., Weissbrod, L. & Zardaryan, D. Introducing Aghitu-3, the first Upper Paleolithic cave site in Armenia. *Aramazd* **2**, 7-23 (2011).

128 Gasparyan, B., Kandell, A. & Montoya, C. (2014).

129 Sablin, M. V. & Khlopachev, G. A. The earliest Ice Age dogs: evidence from Eliseevichi. *Current Anthropology* **43**, 795-799 (2002).

130 Morey, D. F. & Wiant, M. D. Early Holocene domestic dog burials from the North American Midwest. *Current Anthropology* **33**, 224-229 (1992).

131 Acosta, A., Loponte, D. & García Esponda, C. Primer registro de perro doméstico prehispánico (Canis familiaris) entre los grupos cazadores recolectores del humedal de Paraná inferior (Argentina). *Antípoda. Revista de Antropología y Arqueología*, 175-199 (2011).

132 Worthington, B. E. Osteometric Analysis of Southeastern Prehistoric Domestic Dogs. (2008).

133 Ovodov, N. D. *et al.* A 33,000-Year-Old Incipient Dog from the Altai Mountains of Siberia: Evidence of the Earliest Domestication Disrupted by the Last Glacial Maximum. *PLOS ONE* **6**, e22821, doi:10.1371/journal.pone.0022821 (2011).

134 Lee, E. J. *et al.* Ancient DNA analysis of the oldest Canid species from the Siberian Arctic and genetic contribution to the domestic dog. *PloS one* **10**, e0125759 (2015).

135 Pitulko, V., Nikolskiy, P., Basilyan, A. & Pavlova, E. in *Paleoamerican odyssey* 13-44 (2013).

136 Pitulko, V., Pavlova, E. & Basilyan, A. in *Abstract book of the VIth International Conference on mammoths and their relatives. Special Volume SASG.* 155.

137 Pitulko, V., Pavlova, E. & Basilyan, A. Mass accumulations of mammoth (mammoth 'graveyards') with indications of past human activity in the northern Yana-Indighirka lowland, Arctic Siberia. *Quaternary international* **406**, 202-217 (2016).

138 Germonpré, M. *et al.* Palaeolithic and prehistoric dogs and Pleistocene wolves from Yakutia: Identification of isolated skulls. *Journal of Archaeological Science* **78**, 1-19 (2017).
